# A guide to extending and implementing generalized risk-adjusted cost-effectiveness (GRACE)

**DOI:** 10.1007/s10198-021-01367-0

**Published:** 2021-09-08

**Authors:** Darius N. Lakdawalla, Charles E. Phelps

**Affiliations:** 1 School of Pharmacy, Sol Price School of Public Policy, The Leonard D. Schaeffer Center for Health Policy and Economics, University of Southern California, Los Angeles, CA USA; 2 The National Bureau of Economic Research (NBER), Cambridge, MA USA; 3 University of Rochester, Rochester, NY USA; 4 Departments of Economics and Public Health Sciences, University Professor and Provost Emeritus, University of Rochester, Rochester, NY USA

**Keywords:** Quality of life, Life expectancy, Health insurance, Public health insurance, Cost-effectiveness analysis

## Abstract

The generalized risk-adjusted cost-effectiveness (GRACE) model generalizes conventional cost-effectiveness analysis (CEA) by introducing diminishing returns to Health-Related Quality of Life (QoL). This changes CEA practice in three ways: (1) Willingness to pay (WTP) increases exponentially with untreated illness severity or pre-existing permanent disability, and WTP ends up lower for mild diseases but higher for severe diseases compared with conventional CEA; (2) Average treatment effectiveness should be adjusted for uncertainty in outcomes; and (3) The marginal rate of substitution between life expectancy and QoL varies with health state. Implementing GRACE requires new parameters describing risk preferences over QoL, the marginal rate of substitution between life expectancy (LE) and QoL, and the variance and skewness of treatment outcomes distributions. In this paper, we provide: (1) a generalized WTP threshold incorporating the possibility of permanent disability; (2) a simpler method to estimate the tradeoff rate between QoL and LE, eliminating the need to carry out treatment-by-treatment estimates; (3) a more-general method to adjust WTP for illness severity that permits non-constant relative risk-aversion in QoL; (4) a new approach to estimating risk-preferences over QoL, leveraging established empirical methods from “happiness” economics; and (5) a step-by-step guide for practitioners wishing to implement multi-period GRACE analyses.

## Introduction

Lakdawalla and Phelps (hereafter, LP) developed a generalized risk-adjusted cost-effectiveness (GRACE) framework [1, 2] that nests the traditional cost-effectiveness analysis (CEA) framework [3] as a special case. GRACE relaxes the restriction of constant returns to health-related quality of life (QoL) imposed by traditional CEA.^1^ This generalization produces three major implications for the proper conduct of CEA.

First, optimal willingness to pay (WTP) for health improvements increases exponentially as untreated disease severity rises. Cost-effectiveness thresholds become more generous for more severe illness, and lower for milder illnesses, perhaps differing by up to a factor of ten from lowest to highest severity [1]. For similar reasons, GRACE also shows that a given QoL gain is worth more to disabled than to otherwise-similar non-disabled persons.

Second, GRACE incorporates effects of uncertain health outcomes into health technology assessment (HTA). Reductions in outcome uncertainty provide value to risk-averse individuals (and conversely). HTAs should include these effects, particularly in contexts with modest incremental average health gains.

Third, GRACE shows that people with lower QoL are more willing than those in better health to trade remaining life expectancy (LE) for more QoL. Conventional CEA, exemplified by the phrase “a QALY is a QALY…” assumes this tradeoff is independent of baseline QoL, sharply contrasting with population-based surveys [6–8].

In this analysis, we make five new contributions to the GRACE model, all of which help to clarify and broaden its real-world application. First, we generalize previous methods to show why how and why WTP can systematically increase as permanent disability worsens. Next, we simplify methods to estimate the tradeoff between LE and QoL, eliminating the requirement for the treatment-by-treatment estimation proposed by LP [1, 2]. Third, we present a novel method for estimating the risk-preference parameters required by GRACE. Fourth, we widen the range of risk preferences accommodated by the GRACE framework. Finally, we incorporate these findings into a step-by-step guide for practitioners seeking to conduct GRACE studies in both static and dynamic cost-effectiveness value assessments, extending the original static framework [1].

To develop these new ideas, we first summarize the original GRACE model [1, 2], and then present our first four contributions in Sect. “Simplification and expansion of GRACE”. Section “Guide to using the GRACE method” provides a “handbook” for practitioners that clarifies specific implementation steps for using GRACE in a multi-period setting.

## Summary of the GRACE framework

LP consider an individual with expected period utility, $E[U\left(C\right)W\left(H\right)]$, where $C$ is income available for consumption after medical spending is removed, and $H$ is QoL, a stochastic index of health itself (not utility of health). Where all terms are shown in Box 1, the total value of a medical intervention (TVMI) from LP ([1], Eq. 16) is:

1 $$\mathrm{TVMI}=K{\omega }_{H}R\left\{{\mu }_{p}\delta +\phi {p}_{1}{\mu }_{B}\epsilon \right\}.$$

The associated incremental generalized risk-adjusted cost-effectiveness ratio (IGRACER) decision rule is to adopt or reimburse the technology if ([1], Eq. 17):

2 $$\frac{\Delta C}{\left\{{\mu }_{p}\delta +\phi {p}_{1}{\mu }_{B}\epsilon \right\}}\le K{\omega }_{H}R.$$

BOX 1. Definitions $$C=\text{Income}-\text{Medical Spending}=\text{Consumption}$$

$$\varvec{{\Delta C}}=\textbf{Incremental cost of medical technology}$$

$${{\omega_C}=\frac{{U}^{\prime}(C)C}{U\left(C\right)}}=\text{elasticity of utility with respect to C}$$

 $$\mathbf{K=\frac{C}{\omega_C}=}\textbf{ traditional cost-effectiveness threshold}$$

$${{H}_{1S}}=\text{QoL with untreated acute illness and (optionally) disability}$$

$${{\mu }_{H}\equiv E\left({H}_{1S}\right)},\text{average QoL with untreated acute illness and disability}$$

$${B}=\text{stochastic gain in QoL from treatment in the sick state}$$

$${{H}_{0}}=\text{QoL in the baseline period, typically assumed to equal 1}$$

$$\varvec{\omega_H} \;{\mathbf{ = }}\frac{{{\mathbf{W^{\prime}}}\left( {{\mathbf{H}}_{{\mathbf{0}}} } \right){\mathbf{H}}_{{\mathbf{0}}} }}{{{\mathbf{W}}\left( {{\mathbf{H}}_{{\mathbf{0}}} } \right)}}{\textbf{ = elasticity of utility with respect to QoL }}\mathbf{(H)}\textbf{ at }\mathbf{H_0}$$

$${\mathbf{R = }}\frac{{{\mathbf{W^{\prime}}}\left( {{\mathbf{H}}_{{{\mathbf{1S}}}} } \right)}}{{{\mathbf{W^{\prime}(H}}_{{\mathbf{0}}} {\mathbf{)}}}}\textbf{, disease-severity ratio}$$

$${{H}_{0d}}=\text{QoL in the baseline period inclusive of any permanent disability}$$

$${{H}_{1W}}=\text{Health-related QoL in the well state, where }\; {H}_{1W}={H}_{0d}$$

$${{d}^{*}\equiv \frac{{H}_{0}-{H}_{1W}}{{H}_{0}}}=\text{relative quality of life loss from permanent disability}$$

$${{\ell}^{*}\equiv \frac{\left({H}_{0}-{\mu }_{H}\right)}{{H}_{0}}}, \text{average }\%\text{ QoL lost from untreated disease and from disability}$$

$${{t}^{*}\equiv \frac{\left({H}_{0}-E\left({H}_{1S}+B\right)\right)}{{H}_{0}}}, \text{average }\%\text{ QoL lost from treated disease and from disability}$$

$$\varvec{{\mu}_{P}}=\textbf{ Expected gain in probability of survival from treatment}$$

$$\varvec{\delta}=\textbf{marginal rate of substitution (MRS) between life expectancy and QoL}$$

$$\varvec{\phi}=\textbf{probability of acute illness in period 1}$$

$$\varvec{p_1}=\textbf{ probability of survival into period 1}$$

$$\varvec{\mu_B=E\left(B\right)},\textbf{ Expected gain in QoL from treatment}$$

$$\varvec{\epsilon}=\textbf{ Certainty-equivalent QALYs per average QALY gained}$$

^**†**^ $${r}_{H}^{*}=\text{Relative risk-aversion over QoL, evaluated at }{H}_{1S}$$

^**†**^ $${{\pi }_{H}^{*}=}\text{Relative prudence over QoL, evaluated at }{H}_{1S}$$

$${\Delta {\sigma }_{{H}_{T}}^{2}=}\;\left(\text{QoL Variance in Treatment Group}\right)-(\text{QoL Variance in Control Group)}$$

$${\Delta \left[{\gamma }_{1}{\sigma }_{H}^{*3}\right]=}\text{(QoL Skewness in Treatment Group)} - \text{(QoL Skewness in Control Group)}$$

Within Box 1, the parameters appearing directly in Eqs. [(1, 2)] are bolded, and the others are used to calculate the bolded terms.^2^ Most of the Box 1 parameters are common to traditional CEA [3]. Exceptions unique to GRACE include the elasticity of QoL utility (${\omega }_{H})$, the disease-severity ratio $(R)$, the certainty-equivalence ratio $(\epsilon )$, and the marginal rate of substitution between LE and QoL $(\delta )$. LP showed that all these new parameters—except $\delta$—can be calculated using estimates of the variability of treatment outcomes (the $\Delta {\sigma }_{H}$ and $\Delta {\gamma }_{1}{\sigma }_{H}$ terms in Box 1) and of relative risk preferences over health (relative risk-aversion, ${r}_{H}^{*}$ [9] and relative prudence, ${\pi }_{H}^{*}$ [10]). Section “Simplification and expansion of GRACE”C provides empirical methods to estimate these risk-preference parameters.

GRACE nests traditional CEA as the special case of constant marginal utility from QoL. Constant marginal utility in QoL implies that ${\omega }_{H}=R=\epsilon =1$ and ${r}_{H}^{*}=0$. In contrast, GRACE allows for $0<{\omega }_{H}\le 1$, $R\ge 1$, $\epsilon \ne 1$, and ${r}_{H}^{*}\ge 0$. The disease-severity ratio, $R$, adjusts WTP to reflect the effects of diminishing returns to QoL—risk-averse people in worse QoL states place more value on a given QoL gain. The certainty–equivalence ratio, $\epsilon$, adjusts the traditional quality-adjusted life-year (QALY) to account for QoL risk according to consumer risk preferences.

## Simplification and expansion of GRACE

This section develops four improvements to the GRACE framework as originally presented in [1, 2], all of which either extend the range of situations where GRACE can be implemented or simplify its application.

A. Allowing disability in baseline health status
  Begin with one of the most basic ideas in consumer theory—the marginal rate of substitution between two goods. From this notion, we develop a new way to determine WTP for medical technology.
  Consider the cost-effectiveness threshold defined by traditional analysis. Define $${C}_{0}$$ as period zero consumption and $${H}_{1S}$$ as (initially non-stochastic) QoL in the period one sick state. The marginal rate of substitution between $${C}_{0}$$ and $${H}_{1S}$$ measures the period zero WTP for QoL improvement in the sick state. This can be thought of as the WTP for an insurance policy covering treatments that marginally improve QoL in the sick state. The traditional CEA framework presumes $$V\left(C,H\right)=U\left(C\right)W\left(H\right),$$ which yields the marginal rate of substitution (the ratio of marginal utilities of $$C$$ and $$H$$):
  3 $$\frac{{dC_{0} }}{{dH_{{1S}} }} = \frac{{W^{\prime}\left( {H_{{1S}} } \right)U\left( {C_{0} } \right)}}{{U^{\prime}\left( {C_{0} } \right)W\left( {H_{0} } \right)}}.$$
  Traditional CEA imposes the restriction that $$W\left(H\right)=H$$ and thus $${W}^{^{\prime}}\left(H\right)=1$$. It also presumes that $${H}_{0}=1$$. With these restrictions, the traditional cost-effectiveness threshold is $$K\equiv \frac{{C}_{0}}{{\omega }_{C}}$$, where $${\omega }_{C}=\frac{{U}^{^{\prime}}\left({C}_{0}\right)}{U\left({C}_{0}\right)}{C}_{0}$$ is the elasticity of utility with respect to consumption [11]. As discussed in LP ([1], p.3), $$K$$ is the WTP for QoL improvement, and the QALY provides a vehicle for expressing all health improvements—including those resulting from LE gains—in terms of an equivalently valued QoL gain.
  LP relax the restriction that $$W\left(H\right)=H$$ and allow for strictly concave $$W(H)$$. Also, they allow for stochastic health in the sick state: $${H}_{1S}$$ is random. To make the cost-effectiveness threshold non-stochastic, LP evaluate it at $${\mu }_{H}$$, the average QoL in the untreated period 1 sick state. This generalizes Eq. (3) as:
  4 $$\frac{d{C}_{0}}{d{\mu }_{H}}=\left[\frac{{C}_{0}}{{\omega }_{C}{H}_{0}}\right]{\omega }_{H}\left[\frac{{W}^{^{\prime}}({\mu }_{H})}{{W}^{^{\prime}}({H}_{0})}\right].$$
  LP define $${\omega }_{H}\equiv \frac{{W}^{^{\prime}}\left({H}_{0}\right)}{W\left({H}_{0}\right)}{H}_{0}$$, the elasticity of utility with respect to QoL, and $$R\equiv \frac{{W}^{^{\prime}}\left({\mu }_{H}\right)}{{W}^{^{\prime}}\left({H}_{0}\right)}$$, the “disease severity ratio.” With these definitions, the generalized WTP threshold is simply $${K}_{GRACE}=K{\omega }_{H}R$$. LP prove that this is the WTP for a generalized risk-adjusted QALY (GRA-QALY), given by $${\mu }_{p}\delta +\phi {p}_{1}{\mu }_{B}\epsilon$$.
  Here, we further generalize the LP result, allowing for permanent disability. Instead of requiring “excellent” period zero health, we assume instead that period zero health is $${H}_{0d}={H}_{0}(1-{d}^{*})$$, where $$0\le {d}^{*}<1$$ is the percentage of QoL lost to permanent disability, as defined in Box 1.^3^ The earlier LP model and traditional CEA study the special case where $${d}^{*}=0$$. In the more general case, Eq. (4) becomes:
  5 $${K}_{GRACE}=\frac{K{\omega }_{H}R}{{H}_{0}}\left[\frac{W\left({H}_{o}\right)}{W\left({H}_{0d}\right)}\right].$$
  Appendix IA demonstrates that $$\frac{W\left({H}_{0d}\right)}{W\left({H}_{0}\right)}=(1-{d}^{*}\psi$$), where the “disability ratio,” $$\psi$$, is a new parameter that depends on $${\omega }_{H}$$, relative risk-aversion ($${r}_{H}^{*}$$), and relative prudence ($${\pi }_{H}^{*}$$) over QoL, all of which are already required by GRACE analyses. Compactly, the generalized WTP is:
  6 $${K}_{GRACE}=\frac{K{\omega }_{H}R}{{H}_{0}(1-{d}^{*}\psi )}.$$
  Appendix IA demonstrates that $$\psi =1$$ when consumers are risk-neutral over QoL and $$\psi <1$$ when they are strictly risk-averse. Therefore, weak risk-aversion implies $$\psi \le 1$$, which ensures that $${K}_{GRACE}$$ is strictly positive and well-defined.
  Appendix IC proves that people with permanent disabilities (d* > 0) always exhibit greater WTP for QoL improvements than similar non-disabled people, and can sometimes even exhibit weakly greater WTP for LE improvements.^4^ Intuitively, disability lowers the marginal utility of consumption, $${V}_{C}\left(C,{H}_{0d}\right)={U}^{^{\prime}}\left(C\right)W({H}_{0d})$$ and thus increases the willingness to pay for health improvements. This leads to unambiguously higher WTP for QoL among those with disabilities. Some ambiguity arises for LE, because the lower marginal utility of consumption competes with a decline in period utility, $$U\left(C\right)W({H}_{1S})$$, also caused by disability.
  With permanent disability, Eq. (1) for the total value of a medical intervention (TVMI) generalizes to:
  7 $$TVM{I}^{^{\prime}}=\frac{K{\omega }_{H}R}{{H}_{0}(1-{d}^{*}\psi )}\left\{{\mu }_{p}\delta +\phi {p}_{1}{\mu }_{B}\epsilon \right\}.$$
  The total marginal value of life-extension, $${\mu }_{p}$$, is $$\left(\frac{K{\omega }_{H}R}{{H}_{0}(1-{d}^{*}\psi)}\right)\delta$$, which is the WTP for QoL gains $$\left(\frac{K{\omega }_{H}R}{{H}_{0}(1-{d}^{*}\psi )}\right)$$ multiplied by the marginal rate of substitution between LE and QoL. Permanent disability unambiguously increases $$\frac{1}{1-{d}^{*}\psi }$$ and thus pushes up WTP for QoL gains. On the other hand, disability lowers the marginal rate of substitution between LE and QoL, $$\delta$$, because an additional period of life becomes less valuable when period utility falls.^5^
  Appendix IC characterizes the net effect: defining $$B$$ as the stochastic QoL benefit of medical technology, permanent disability weakly increases WTP for LE if and only if $$\frac{E\left(W\left({H}_{1S}+B\right)\right)}{W\left({H}_{0d}\right)}$$ rises with disability. In other words, if a given disability lowers utility proportionally more in healthy states than sick states, it will increase the WTP for LE; heuristically, this requires that acute illness “crowds out” some of disability’s QoL effects. For example, this will be true if utility exhibits CRRA and disability lowers QoL by a constant percentage in both healthy and sick states.
  To characterize the condition more generally, define $${t}^{*}$$ as the percentage QoL loss after treatment, inclusive of disability, so that $$E\left({H}_{1S}+B\right)={H}_{0}\left(1-{t}^{*}\right)$$, as in Box 1. Appendix IC proves that—under CRRA utility—disability weakly increases WTP for LE if and only if $$\frac{\partial {t}^{*}}{\partial {d}^{*}}\le \frac{1-{t}^{*}}{1-{d}^{*}}$$. This is always satisfied for curative therapies where $${t}^{*}={d}^{*}$$, because disability reduces QoL by the same percentage in both states. Imperfect therapies make this restriction non-trivial.
  This analysis explains why standard methods of CEA discriminate against disabled persons, since the standard approach assumes $${d}^{*}=0$$. Holding $${d}^{*}=0$$ implies that a QALY is *always* worth less for disabled persons than for otherwise-similar non-disabled persons. This problem has led to several ad hoc attempts to resolve this inconsistency [12, 13]. Such ad hoc adjustments are unfounded and perhaps even unnecessary. Under GRACE, disability always increases the WTP for QoL gains and can weakly increase the WTP for LE gains if $$\frac{\partial {t}^{*}}{\partial {d}^{*}}\le \frac{1-{t}^{*}}{1-{d}^{*}}$$. Analysts can use this condition to determine the frequency with which disability leaves WTP for LE at least unchanged. The U.S. Affordable Care Act forbids use of CEA methods that discount the value of life for disabled people, both for assessing treatment value and determining Medicare coverage.^6^ GRACE demonstrates how WTP for life-extension can rise with disability and may help provide a framework for conducting CEA compatible with current law.
B. Simplified estimation of the marginal rate of substitution (MRS)
  We now turn to a second improvement upon LP, showing how to recover the marginal rate of substitution, $$\delta$$, from other GRACE parameters. This obviates the need for disease-specific estimation of $$\delta$$. LP formally defined this MRS as:
  8 $$\delta \equiv {\text{ }}\frac{{\phi \left[ {E\left[ {W\left( {H_{{1S}} + B} \right)} \right] + \left( {1 - \phi } \right)W\left( {H_{{1W}} } \right)} \right]}}{{W^{\prime}\left( {\mu _{H} } \right)}} \equiv {\text{ }}\frac{V}{{W^{\prime}\left( {\mu _{H} } \right)}}.$$
  Recall $${H}_{1S}$$ is the stochastic period 1 level of untreated health in the “sick” state, $$B$$ is the stochastic QoL benefit arising from medical interventions, and $${H}_{1W}$$ represents non-stochastic health when no acute illness occurs. The numerator, $$V$$, is the gain in expected utility produced by the marginal increase in survival probability, and the denominator, $$W^{\prime}(\mu _{{\text{H}}} )$$, describes the marginal gain in utility arising from improved QoL in the untreated sick state. Since disability influences $${\mu }_{H}$$, $${H}_{1S}$$, and $${H}_{1W}$$, $$\delta$$ varies with disability. For the same reason, $$\delta$$ varies with the severity of untreated illness $$\left({\mu }_{H}\right)$$. This is why LP specified that $$\delta$$ must be estimated on a disease-by-disease basis.
  Appendix IB proves that $$\mathrm{TVMI}$$ in Eq. (1) can also be written as^7^:
  9 $$\mathrm{TVMI}=K\left[{\mu }_{p}\rho {H}_{0}+\phi {p}_{1}{\omega }_{H}R{\mu }_{B}\epsilon \right].$$
  The new parameter, $$\rho \equiv \frac{\phi \left[E\left[W\left({H}_{1S}+B\right)\right]+\left(1-\phi \right)W\left({H}_{1W}\right)\right]}{\mathrm{W}({\mathrm{H}}_{0})}=\frac{V}{W\left({H}_{0}\right)},$$ is the ratio of period 1 expected QoL utility to baseline period QoL utility.
  In general, $$0<\rho \le 1$$, where equality holds only for perfect cures coupled with $${d}^{*}=0$$. Furthermore, $${H}_{1W}={H}_{0}(1-{d}^{*})$$ and $$E({H}_{1S})={H}_{0}(1-{\ell}^{*})$$, where $${\ell}^{*}$$ is the average percentage health loss from the acute illness and any pre-existing permanent disability (see Box 1).^8^ Both $${H}_{1W}$$ and $${H}_{1S}$$ are measured on a [0, 1] scale for an appropriate index of health-related QoL.
  We now show that $$\rho$$ can be estimated simply from standard measures of treatment effectiveness, such as from randomized clinical trials (RCTs) or similar evaluation methods, combined with measures of risk-preferences that are not disease-specific. This implies that $$\delta$$ can be estimated without disease-specific preference studies.
  Appendix IB demonstrates that $$\delta =\frac{\rho {H}_{0} }{{\omega }_{H}R}$$, using the definitions of $$\rho$$, $$\delta$$, and $${\omega }_{H}$$. Moreover, $$\rho$$ can be expressed using Taylor series expansions around $${H}_{0}$$, the baseline health level. Appendix IB describes $$\rho$$ as the $$\phi$$-weighted sum of two parts, one for the “sick” (acute illness) state $$(S)$$ and the other for the “well” state $$(W)$$, both of which should incorporate effects of any permanent disabilities:
  10 $$\rho =\phi {\rho }_{S}+\left(1-\phi \right){\rho }_{W}$$
  Now define $${t}^{*}$$ such that $$E\left({H}_{1S}+B\right)={H}_{0}\left(1-{t}^{*}\right)$$, so $${t}^{*}$$ is the average percentage reduction in QoL *after* treatment. For example, if *ex ante* health is perfect $$({H}_{0}=1)$$, and average post-treatment QoL is 0.75, then $${t}^{*}=0.25$$. If the treatment is perfect, then $${t}^{*}={d}^{*}$$, and if $${d}^{*}=0$$, then $${t}^{*}=0$$ also.
  Appendix IB shows that:
  11 $${\rho }_{S}=1-{\omega }_{H}\left[{t}^{*}+\frac{1}{2}{r}_{H}^{*}{t}^{*2}+\frac{1}{6}{r}_{H}^{*}{\pi }_{H}^{*}{t}^{*3}+\dots \right].$$
  In Eq. (11), health losses are “discounted” by $${\omega }_{H}$$, which reflects rates of diminishing returns to health. Similarly, the full Taylor Series expansion for the “well state” portion of $$\rho$$ is:
  12 $$\rho_W=1-\omega_H\left[d^*+\frac{1}{2}r^*_H {d^*}^2+\frac{1}{6}\pi_H^*r_H^*{d^*}^3+\dots \right]
$$
  Thus, $${\rho }_{W}=1$$ when $${d}^{*}=0$$. Equation (12) completes the characterization of Eq. (10). Once the utility parameters $${\omega }_{H}, {r}_{H}^{*}\; \mathrm{and}\; {\pi }_{H}^{*}$$ are estimated, $${t}^{*}$$ and $${d}^{*}$$ can be estimated from standard QoL outcomes studies. With these new results, analysts can estimate $$\rho$$ without the need of disease- or treatment-specific studies that would otherwise be required when using the definition of $$\delta$$ in Eq. (8).
C. Estimating willingness to pay for non-constant relative risk-aversion
  LP demonstrate that WTP for generalized risk-adjusted QALYs is $${K}_{GRACE}=K{\omega }_{H}R$$. Prior research demonstrates that $$K=\frac{C}{{\omega }_{C}}$$ [11]. The general economics literature suggests that $${0.3\le \omega }_{C}\le 0.5$$ [11, 14, 15]. This implies (approximately) that for consumption level $$C$$, $$2C\le K\le 3C$$.
  In general, $$0<{\omega }_{H}\le 1$$ and $$R\ge 1,$$ where $$R$$ grows with illness severity. LP showed how to recover estimates of $$R$$ under the assumption of constant relative risk-aversion (CRRA) over QoL. We next explain why and how CRRA may incorrectly restrict possible values of $$R$$, and then present a more-general method for estimating $$R$$ under non-CRRA utility.
  1. WTP under constant relative risk-aversion
    Table 1 gives a representative set of values for $$R$$ under CRRA utility and different combinations of $${r}_{H}^{*}$$ and untreated illness severity ($$\ell^{*}$$).

**Table 1:** Values of disease-severity multiplier (R) by disease severity ${(\ell}^{*})$ and relative risk-aversion $({r}_{H}^{*})$

	*r*_H_^*^				
ℓ*	0	0.25	0.5	0.75	1
0	1	1	1	1	1
0.1	1	1.03	1.05	1.08	1.11
0.3	1	1.09	1.2	1.31	1.43
0.5	1	1.19	1.41	1.62	2
0.7	1	1.35	1.83	2.47	3.33
0.9	1	1.78	3.15	5.61	10
    Table 1 illustrates how willingness-to-pay for QoL improvements rise under CRRA as both relative risk aversion ($${r}_{H}^{*})$$ and severity of illness $$({\ell}^{*})$$ increase.^9^
    To pursue Table 1’s implications, note that traditional CEA presumes that ${\omega }_{H}=1,$ and ${r}_{H}^{*}=0$. For illustrative purposes, assume that ${\omega }_{C}=0.33$, a value within the range of prior empirical literature [11, 14, 15]. In this case, $K=3C$ at all levels of disease severity. For example, at annual consumption of $C=\$\mathrm{50,000}$, the WTP per QALY under traditional CEA would be $K=\$\mathrm{150,000}$ for all disease levels.
    By comparison, ${K}_{GRACE}=\frac{K{\omega }_{H}R}{{H}_{0}(1-{d}^{*}\psi )}.$ For simplicity, let ${d}^{*}=0$ and ${H}_{0}=1$. Now, suppose that ${\omega }_{H}=0.5$ instead of 1.0, and ${\omega }_{C}$ remains as 0.33. Thus, $K{\omega }_{H}=1.5C=\$\mathrm{75,000}$. To calibrate $R$, note that ${\omega }_{H}=1-{r}_{H}^{*}$ under CRRA, and thus ${\omega }_{H}=0.5$ implies ${r}_{H}^{*}=0.5.$^10^ From Table 1, ${r}_{H}^{*}=0.5$ implies values for $R$ ranging from 1.0 (for $\ell^*\approx 0$) to 3.15 (for ${\ell}^{*}=0.9)$. This means that $\$\mathrm{75,000}\le {K}_{GRACE}\le \$\mathrm{236,250}.$ Similarly, when ${\omega }_{H}=0.75 \text{ (hence }{r}^{*}=0.25 \text{ with CRRA})$, ${\$\mathrm{112,500}\le K}_{GRACE}\le \$\mathrm{200,270}.$ When ${\omega }_{H}=0.25$ (hence ${r}_{H}^{*}=0.75$), ${\$\mathrm{37,500}\le K}_{GRACE}\le \$\mathrm{210,377}.$ If included, permanent disability $({d}^{*}>0)$ would increase WTP relative to these estimates.
    As these examples demonstrate, the severity gradient of $R$ rises as ${r}_{H}^{*}$ rises. At the same time, however, the tight linkage between ${\omega }_{H}$ and ${r}_{H}^{*}$ imposed by CRRA restricts the space of possible values for ${r}_{H}^{*}$. The implications of non-CRRA utility range from minimal to consequential, depending on specific parameter estimates. We pursue this issue next.
  2. Relaxing the CRRA assumption
    The Hyperbolic Absolute Risk Aversion (HARA) family of utility permits all forms of risk-aversion, including not only CRRA, but also increasing and decreasing relative risk aversion (IRRA and DRRA).^11^ We use a slightly simplified HARA utility function (see Appendix II):
    13 $$U\left(H\right)=\left[\frac{1-\gamma }{\gamma }\right]{Z}^{\gamma },$$
    where $Z=H+\eta$. The parameter $\eta$ has the same units of measurement as $H$, and can be positive or negative. We restrict the domains of $H$ and $\eta$ such that $Z\ge 0$, and we specify that $U\left(H\right)=0 \text{ for }Z=0$. For this simplified HARA model, Appendix II proves that:
    14 $${r}_{H}^{*}=\left(1-\gamma \right)\left(\frac{H}{Z}\right)=\left(1-\upgamma \right)\left[\frac{H}{H+\eta }\right].$$
    When $\eta =0$, HARA simplifies to CRRA utility, because $H=Z$ and thus ${r}_{H}^{*}=(1-\gamma$), which is constant. However, when $\eta >0$, ${r}_{H}^{*}<(1-\gamma )$, and when ${\eta <0, r}_{H}^{*}>(1-\gamma )$, and can exceed 1.0 for sufficiently small values of $\gamma$ and sufficiently large absolute values of $\eta$.^12^ This expands the realm of possible values of ${r}_{H}^{*}$ beyond those available with the CRRA restriction.
    Similarly (see Appendix II), the elasticity of utility with respect to $H$ is:
    15 $${\omega }_{H}=\gamma \left[\frac{H}{H+\eta }\right]=\gamma \left(\frac{H}{Z}\right).$$
    Taking the ratio of (14) to (15) yields an important insight: Since $\left(\frac{H}{Z}\right)$ cancels out:
    16 $$\frac{{\mathrm{r}}_{H}^{*}}{{\omega }_{H}}=\frac{1-\gamma }{\gamma }.$$
    Therefore,
    17 $$\gamma =\frac{{\omega }_{H}}{{r}_{H}^{*}+{\omega }_{H}}.$$
    Hence, $0<\gamma <1$ since both ${r}_{H}^{*}\; \mathrm{and} \;{\omega }_{H}$ are positive for risk-averse individuals.
    Identifying $\gamma$ also provides a basis for estimating *all* higher-order risk parameters in HARA utility once ${r}_{H}^{*}\text{ and }{\omega }_{H}$ (and hence $\gamma )$ have been estimated. Phelps and Cinatl [15] prove that for HARA utility:
    18 $${\pi }^{*}=\frac{2-\gamma }{1-\gamma }{r}^{*}.$$
    and:
    19 $${\tau }^{*}=\left[\frac{3-\gamma }{1-\gamma }\right]{r}^{*}.$$
    The general form, where ${\zeta }_{j}^{*}$ is the *j*th relative risk preference parameter, is:
    20 $${\zeta }_{j}^{*}=\frac{j-\gamma }{1-\gamma }{r}^{*}.$$
    These higher-order risk parameters enter the Taylor-Series expansions for the severity-of-illness ratio $R$, the disability ratio $\psi$, and the estimation of $\rho$ outlined above, so having a convenient way to estimate them is useful. This generalizes previous methods that relied upon CRRA to estimate $R$ from ${\ell}^{*}$.^13^ With these derivations for HARA utility, employing GRACE requires only two QoL parameters—${\omega }_{H}\mathrm{ and }{r}_{H}^{*}$—the estimation of which we turn to in the next section.
    Appendix III illustrates that higher-order relative risk preference parameters may be underestimated by 50% or more for some HARA utility functions that fail to satisfy CRRA. Appendix III also shows that: (1) relative bias is exacerbated if the degree of relative risk-aversion is lower; and (2) bias becomes more consequential if QoL outcomes possess highly non-normal distributions, in which case relative prudence and other higher-order risk parameters become more important.^14^ These results further emphasize the importance of understanding whether utility is CRRA or not, and if not, properly estimating the relative risk preference parameters.
D. Parameter estimation using “happiness” models

LP [1] proposed estimating relative risk preference parameters using discrete choice experiments (DCEs) of the sort that prior research [16] has employed for recovering relative risk preferences over consumption. This method remains viable. Once analysts have estimated ${r}_{H}^{*}$ and ${\pi }_{H}^{*}$ using standard DCE methods, they can exploit the properties of HARA utility to recover $\gamma$ [see Appendix Eq. (72)], and they can use Eq. (16) to recover ${\omega }_{H}.$ Best-practices for the design and conduct of discrete choice experiments have been presented elsewhere [17].

However, discrete choice approaches tend to rely on relatively complex survey instruments. In some cases, analysts may wish to field shorter and simpler survey instruments to reach larger samples of respondents and achieve higher completion rates. For such contexts, we offer an additional strategy that exploits the empirical “happiness” literature pioneered by Easterlin [18]. Specifically, we show how to use the empirical happiness approach to estimate ${\omega }_{H}$ and ${r}_{H}^{*}$, under the assumption of HARA utility. Analysts can then exploit results of Sect. “Simplification and expansion of GRACE”C to recover the full set of risk preference parameters from ${\omega }_{H}\text{ and }{r}_{H}^{*}$, estimating $\gamma$ using Eq. (17) and higher-order terms using Eqs. (18, 19, 20).

Following Easterlin [18], we propose that analysts estimate “utility” by using respondents’ self-reported happiness on an ordinal scale (“*Happy”)*, and the use of higher-order regression terms to capture the curvature of preferences more fully. Furthermore, we propose the collection of data on respondents’ annual consumption $(C$) and some suitable index of health-related QoL $(H)$. Now, exploiting the “translog utility” estimation approach of Christensen, Jorgenson and Lau [19], consider the following regression model relating self-reported happiness to self-reported levels of health $({H}_{i})$ and consumption $({C}_{i})$ for individual $i$:

21 $$\mathrm{ln}\left(Happ{y}_{i}\right)\;=\;{\beta }_{1}\mathrm{ln}\left({H}_{i}\right)+\frac{1}{2}{\beta }_{2}{\left(\mathrm{ln}\left({H}_{i}\right)\right)}^{2}+{\beta }_{3}\mathrm{ln}\left({C}_{i}\right)+\frac{1}{2}{\beta }_{4}{\left(\mathrm{ln}\left({C}_{i}\right)\right)}^{2}+{\epsilon }_{i}.$$

Suppressing individual-specific subscripts, $i$, Eq. (21) provides a second-order approximation of any sufficiently differentiable utility function [19]. The above formulation suppresses interaction terms between $\mathrm{ln}(C)$ and $\mathrm{ln}(H)$, but these could be added if desired. In what follows, we develop our results in terms of $H$, but exactly analogous developments can be made to recover relative risk-aversion parameters over consumption, $C$.

Noting that $\frac{\partial \mathrm{ln}\left(Happy\right)}{\partial \mathrm{ln}\left(H\right)}={\omega }_{H}$, from Eq. (21):

22 $${\omega }_{H}\left(H\right)={\beta }_{1}+{\beta }_{2}\mathrm{ln}\left(H\right).$$

This implies the estimator, $\widehat{{\omega }_{H}}\left(H\right)=\widehat{{\beta }_{1}}+\widehat{{\beta }_{2}}\mathrm{ln}(H)$, so ${\omega }_{H}$ varies with $H$. Equation (16) then implies that ${r}_{H}^{*}$ will vary with $H$. And, Eqs. (18, 19, 20) then imply that ${\pi }_{H}^{*}$ and the other higher-order risk-preference parameters will vary with $H$ also. Therefore, this estimation framework allows risk preferences to vary with the level of health $(H)$, instead of requiring CRRA.

We now turn to estimation of ${r}_{H}^{*}$ using the “happiness” equation approach, details of which appear in Appendix IV. To begin, we apply a general rule for any sufficiently differentiable utility function. Define ${\epsilon }^{\omega }\equiv \frac{\partial {\omega }_{H}}{\partial H}\frac{H}{{\omega }_{H}}$ as the elasticity of ${\omega }_{H}$ with respect to $H$. Suppressing the various dependencies on $H$, Appendix IV proves that:

23 $${\epsilon }^{\omega }=1-\left({r}_{H}^{*}+{\omega }_{H}\right).$$

So,

24 $${r}_{H}^{*}=\left(1-{\omega }_{H}\right)-{\epsilon }^{\omega }.$$

When utility is CRRA, ${\epsilon }^{\omega }=0$. Thus, Eq. (24) implies that ${\omega }_{H}=1-{r}_{H}^{*}$ and ${0<r}_{H}^{*}<1.$ When utility has declining relative risk aversion (DRRA), Eq. (16) implies that ${\epsilon }^{\omega }<0$. Thus, Eq. (23) implies that ${r}_{H}^{*}+{\omega }_{H}>1$. The converse is true if utility exhibits increasing relative risk aversion (IRRA).

With an estimate of ${\omega }_{H}$ in hand, we can use Eq. (24) to recover ${r}_{H}^{*}$, provided we can estimate ${\epsilon }^{\omega }.$ Note that since ${\omega }_{H}={\beta }_{1}+{\beta }_{2}\mathrm{ln}(H)$, then $\frac{\partial {\omega }_{H}}{\partial H}=\frac{{\beta }_{2}}{H}$. From this,

25 $${\epsilon }^{\omega }\left(H\right)=\frac{\partial {\omega }_{H}}{\partial H}\left(\frac{H}{{\omega }_{H}}\right)=\frac{{\beta }_{2}}{{\omega }_{H}}=\frac{{\beta }_{2}}{{\beta }_{1}+{\beta }_{2}\mathrm{ln}\left(H\right)}.$$

The empirical analog, $\widehat{{\epsilon }^{\omega }}(H)=\frac{{\widehat{\beta }}_{2}}{{\widehat{\beta }}_{1}+{\widehat{\beta }}_{2}\mathrm{ln}\left(H\right)}$, varies with $H \text{ unless }\widehat{{\upbeta }_{2}}=0$. With estimates of ${\omega }_{H}$ and ${\epsilon }^{\omega }$ in hand, we can now recover ${r}_{H}^{*}$ from Eq. (24) as:

26 $$\widehat{{r}^{*}}\left(H\right)=1-{\widehat{\omega }}_{H}\left(H\right)-\widehat{{\epsilon }^{\omega }}\left(H\right)=1-\left({\widehat{\beta }}_{1}+{\widehat{\beta }}_{2}\mathrm{ln}\left(H\right)\right)-\frac{{\widehat{\beta }}_{2}}{{\widehat{\beta }}_{1}+{\widehat{\beta }}_{2}\mathrm{ln}\left(H\right)}.$$

If ${\beta }_{2}<0$ (DRRA utility) then ${r}_{H}^{*}$ can exceed $\left(1-{\widehat{\omega }}_{H}\right)$. For relatively small values of $\widehat{{\beta }_{1}}$ and sufficiently large negative values of $\widehat{{\beta }_{2}}$, $\widehat{{r}_{H}^{*}}$ can exceed 1.0. This widens the potential range for values of $\widehat{{r}^{*}}\left(H\right)$ compared with the stricter requirements of CRRA utility.

Using estimates of ${\omega }_{H}^{*}$ and ${r}_{H}^{*}$, we can return to the HARA utility structure (Section “Simplification and expansion of GRACE”C2) to estimate $\gamma$ [using Eq. (17)] and all higher-order relative risk preference parameters [using Eqs. (18, 19, 20)]. In turn, analysts can estimate $R$ using these parameters and the formula for *R* from ([1], Eq. 19), reproduced here for convenience:

27 $$R=\left\{1+{r}_{H}^{*}{\ell}^{*}+\frac{1}{2}{r}_{H}^{*}{\pi }_{H}^{*}{{\ell}^{*}}^{2}+\frac{1}{6}{r}_{H}^{*}{\pi }_{H}^{*}{\tau }_{H}^{*}{{\ell}^{*}}^{3}+\dots .\right\}.$$

This method enables more robust estimates of $R$, compared with Table 1, which relies on the restrictive CRRA assumption.

## Guide to using the GRACE method

GRACE calls for practitioners to introduce new information into HTAs. This section presents a guidebook for carrying out these tasks. It includes two sections—things that must be done only once, and things that must be done for every new HTA.

A. Things done only once
  In the language of biopharmaceutical companies, these are “above-brand” tasks. They estimate parameters describing people’s attitudes towards QoL risk. Practitioners who analyze specific healthcare interventions need not perform these tasks for each new HTA.
  1. One-time parameter estimates
    These “one-time” parameters represent a full complement of measures characterizing consumer attitudes towards QoL risk. They include:
    1. The elasticity of utility with respect to health (${\omega }_{H})$. Conceptually, $0<{\omega }_{H}<1$ if marginal utility from improved health is positive but declining.
    2. Relative risk aversion ${(r}_{H}^{*}$), related to variance of treatment outcomes. This parameter determines how the marginal utility of QoL changes as QoL itself changes.
    3. Relative prudence $({\pi }_{H}^{*})$, related to skewness of treatment outcomes. This parameter determines how ${r}_{H}^{*}$ changes as $H$ changes.
    4. Optionally, relative temperance ${(\tau }_{H}^{*})$, related to excess kurtosis of treatment outcomes. This parameter determines how ${\pi }^{*}$ changes as $H$ changes.
    Technically, LP rely on relative risk-aversion parameters evaluated at ${\mu }_{H}$, the average QoL in the untreated sick state, which will vary across disease states.^15^ Under CRRA, this subtlety can be ignored. However, analysts wishing to relax the CRRA assumption would ideally estimate relative risk preferences for varying degrees of QoL to create a library of such estimates applicable to a range of diseases.
    Section “Simplification and expansion of GRACE”D (along with Appendices II and IV) presents two methods for recovering relative risk preference parameters: (1) estimating ${r}_{H}^{*}$ and ${\pi }_{H}^{*}$ via discrete choice experiments and recovering the remaining parameters; or (2) estimating ${\omega }_{H}\text{ and }{r}_{H}^{*}$ from “happiness economics” regression parameters and features of HARA utility functions.
  2. Estimating severity-adjusted WTP
    Estimating risk- and severity-adjusted WTP requires the preference parameters described above and estimates of disease severity. Recall from Sect. “Simplification and expansion of GRACE”A that in the conventional model, where $C$ is annual consumption and ${\omega }_{C}$ is the elasticity of utility with respect to C [11]:
    28 $$K=\frac{C}{{\omega }_{C}}.$$
    In the GRACE framework:
    29 $${K}_{GRACE}=\frac{K{\omega }_{H}R}{{H}_{0}\left(1-\psi{d}^{*} \right)}.$$
  In addition to the measure of health loss from disability, d*, this adds three new risk parameters—${\omega }_{H}$, $R$ and the new parameter $\psi ,$ which can be estimated using risk parameters already required (see Appendix IA). As shown in Eq. (10), the parameter $R$ depends on relative risk preferences, and the severity of untreated disease (${\ell}^{*})$. “Happiness economics” methods (Section “Simplification and expansion of GRACE”D) permit estimation of the key parameters ${\omega }_{H}$ and ${\omega }_{C}$ from the same data set, if desired.
  Severity (untreated QoL) is already estimated for many diseases of interest [20]. We presume that, over time, tables reporting disease severity for various diseases (${\ell}^{*})$ will be created by public payers or health technology assessment bodies, such as NICE in the UK or CMS in the US. For example, just as CMS has calculated hospital payment levels using Diagnosis-Related Groups, it may wish to measure disease-severity within a similar taxonomy. Those disease-severity values, combined with population estimates of risk-preferences, allow estimation of $R$, ${\omega }_{H}$, and ultimately the “system-wide” threshold of ${K}_{GRACE}=\frac{K{\omega }_{H}R}{{H}_{0}\left(1-\psi{d}^{*} \right)}$, for various levels of disease and disability severity. These are “done once” and need not be undertaken by practitioners evaluating specific medical interventions.
  In standard CEA, medical technologies are considered “welfare-improving” if incremental costs ($\Delta Cost)$ relative to incremental benefits $({\Delta} \text{QALYs})$ are less than society’s WTP, normally expressed as:
  30 $$K \equiv \frac{C}{{\omega _{C} }} \ge \frac{{\Delta {\text{Cost}}}}{{\Delta {\text{QALYs}}}}.$$
  GRACE modifies this so that:
  31 $${K}_{GRACE}\equiv \frac{K{\omega }_{H}R}{{H}_{0}\left(1-\psi{d}^{*} \right)}\ge \frac{\Delta Cost}{\Delta \left(\text{GRA-QALYs}\right)},$$
  where the generalized risk-adjusted QALY (GRA-QALY) is $\left\{{\mu }_{p}\delta +\phi {p}_{1}{\mu }_{B}\epsilon \right\}$.
B. Parameters that must be estimated for each new treatment
  Both $R$ and GRA-QALYs themselves contain parameters that vary by treatment intervention, listed next:
1. *The average incremental gain in QALYs compared with the current “best practice treatment.* GRACE calls this ${\mu }_{B}$ (“B” for “Benefit”). Current studies routinely estimate ${\mu }_{B},$ the difference between average QoL outcomes for the new treatment (T) and its comparison (C).
2. *The variance of patient outcomes, both for “T” and “C.”* GRACE uses *the difference* between these variances, described in the model as $\Delta {\sigma }^{2}\equiv {\sigma }_{{H}_{T}}^{2}-{\sigma }_{{H}_{C}}^{2}$, where ${\sigma }^{2}_{{H}_{T}}$ and ${\sigma}^{2}_{H_C}$ are the QoL variance in the treatment and comparator groups. Reductions in variances of treatment options provide additional value beyond mean health gains, and conversely.
3. *The skewness of patient outcomes, both for “T” and “C”.* This requires estimating Pearson’s skewness parameters, ${\gamma }_{1{H}_{T}}$ and ${\gamma }_{1H_C}$, for both treatment and comparator populations.^16^ Where (generally) ${\sigma }^{3}={\left[{\sigma }^{2}\right]}^\frac{3}{2}$, GRACE uses $\Delta Skewness=\Delta \left[\gamma {\sigma }^{3}\right]\equiv {\gamma }_{1{H}_{T}}{\sigma }_{{H}_{T}}^{3}-{\gamma }_{1{H}_{C}}{\sigma }_{{H}_{C}}^{3}$, where the subscripts ${H}_{T} \text{ and }{H}_{C}$ represent (respectively) the treatment and comparator populations. Increases in positive skewness add value for any given change in variances of outcomes (and conversely).
4. *Optionally, the kurtosis of outcomes in treatment and comparison groups.* Kurtosis magnifies effects of variance, so reductions in excess kurtosis have independent value in addition to other components of value in GRACE. GRACE uses the difference in kurtosis values, parallel to the measures of skewness. Estimating kurtosis requires large sample populations, potentially infeasible in some clinical studies.^17^
5. *Estimates of the relative QoL loss from disease, both before and after treatment.* GRACE measures these on a percentage basis, relative to *ex ante* health states that obtain before the illness occurred.
  - *Loss in untreated states*: This parameter ${\ell}^{*}$ (defined in Box 1) represents percentage losses in QoL suffered in the untreated states. This includes the QoL effects of acute illness and of any permanent disabilities. Diseases with no QoL consequences would imply ${\ell}^{*}=0$; one that lowers QoL by 50% from its baseline state would involve ${\ell}^{*}=0.5$, and so on. Many estimates of ${\ell}^{*}$ are already available from CEA studies [21]. This parameter is disease-specific, but not treatment-specific.
  - *Loss in treated states:* This parameter ${t}^{*}$ (defined in Box 1) reflects percentage losses in QoL remaining after diseases are treated. As with ${\ell}^{*}$, this includes the QoL effects of treated acute illness and of any permanent disabilities. If treatment always completely cures patients, ${t}^{*}=0$.^18^ If post-treatment states feature QoL that lies 30% below the *ex ante* (pre-disease) QoL level $({H}_{0})$, then ${t}^{*}=0.3$, etc*.* These values must be estimated for each disease/treatment pair. As explained in Sect. “Simplification and expansion of GRACE”B, they are used to estimate the MRS between QoL and LE in GRACE.
  - *Permanent disability loss:* This parameter ${d}^{*}$ (defined in Box 1) reflects the percentage loss in QoL created by permanent disabilities that predated the arrival of the period one acute illness. This is an optional term that analysts may wish to consider when studying health interventions among people with disabilities. Under GRACE, people with disabilities always exhibit higher WTP for QoL improvements and sometimes exhibit equal or higher WTP for LE gains (see Sect. “Simplification and expansion of GRACE”A).
  - *Ex ante QoL level:* ${H}_{0}$ measures QoL prior to onset of relevant disease in populations of interest. Typically, analysts will presume ${H}_{0}=1$.
6. *The average increase in survival probability to period 1, compared with the control therapy, described in GRACE as *${{\varvec{\mu}}}_{{\varvec{p}}}$**.** The approach to calculating long-term survival gains differs depending on the stationarity of QoL distributions; we provide guidance on this issue in Sect. “Guide to using the GRACE method”C below.
7. *The probability of surviving to period 1, described in GRACE as* ${{\varvec{p}}}_{1}$. This is the level of survival probability, rather than its change.
8. *The population incidence of the disease of interest,* ${\varvec{\phi}}$. This parameter is typically estimated in epidemiological burden of illness studies.
  Traditional cost-effectiveness and comparative-effectiveness studies estimate Parameter 1, the mean gain in QoL due to treatment. Nearly all such studies can be used to recover Parameters 2, 3, and 4, simply by recording variance, skewness, and kurtosis of QoL outcomes for patients in treatment and control groups. Such studies likely already produce estimates for parameters listed in 5, which concern QoL levels in treated, untreated, and pre-onset states. The same is true for survival parameters 6 and 7, also routinely estimated in current cost-effectiveness studies. Finally, existing burden of illness studies likely already estimate disease incidence, parameter 8.
  The risk-preference parameters from Sect. “Guide to using the GRACE method”A.1 and estimates of ${\ell}^{*}$ (item 5a in the list above) together allow estimates of $R$ using Eq. (10). Next, the list of intervention-specific parameters above, combined with risk-preference parameters in Sect. “Guide to using the GRACE method”A, imply intervention-specific values of $\rho$, which can be calculated using Eqs. (23, 24, 25) above.
  Next, parameters in Sect. “Guide to using the GRACE method”A and the list above imply intervention-specific values of $\epsilon$, which can be calculated from ([1], Eq. 9), reproduced here:
  32 $$\upepsilon \approx 1+\left[\frac{1}{{\mu }_{B}}\right]\left[-\frac{1}{2}{r}_{H}^{*}\left(\frac{1}{{\upmu }_{\mathrm{H}}}\right)\Delta {\upsigma }_{\mathrm{H}}^{2}+\frac{1}{6}{\pi }_{H}^{*}{r}_{H}^{*}{\left(\frac{1}{{\upmu }_{\mathrm{H}}}\right)}^{2}\Delta \left[{\gamma }_{1}{\sigma }_{H}^{3}\right]+\dots \right].$$
  When there are no differences in variance and skewness of QoL in the treatment and comparator groups, then $\epsilon =1$, and no need exists to adjust mean treatment benefits. Improvements (worsening) of variance add to (subtract from) overall benefit, and increases (decreases) in skewness add to (subtract from) mean benefits.
C. Estimating net monetary benefit and cost-effectiveness
  The original GRACE formulation employed a static two-period setting, in which patients are healthy in period zero and potentially sick in period one. Nonetheless, this static framework can handle dynamic treatment contexts and models (e.g., Markov models). We demonstrate this for two different cases.
  Begin with the more-general case in which the means, variances, and/or skewness in QoL outcomes vary over time. Consider an assessment of value over periods $n=\mathrm{1,2},\dots ,N$. The following parameters would then need to be estimated on a period-by-period basis: ${\mu }_{Bn}$ (mean QoL benefit in period $n$), $\Delta {\sigma }_{Hn}^{2}$ (difference in the variance of QoL benefit in period $n$), $\Delta {\left[\gamma {\sigma }_{H}^{3}\right]}_{n}$ (difference in skewness of QoL benefit in period $n$), ${\ell}_{n}^{*}$ (percentage QoL loss from untreated disease in period $n$, and ${t}_{n}^{*}$ (percentage QoL loss from treated disease in period $n$).^19^ Moreover, since $\rho$, $\delta$, and $\epsilon$ also depend on treatment outcomes, they would be indexed as ${\rho }_{n}$, ${\delta }_{n}$, and ${\epsilon }_{n}$. Since $R$ depends on ${\ell}^{*}$, which might vary over time, it too is indexed as ${R}_{n}$. Define $\Delta {C}_{n}$ as incremental treatment cost incurred in period $n$, which is equivalent to the incremental reduction in non-health period $n$ consumption, ${C}_{n}$. Finally, define ${\mu }_{pn}$ as the average increase in the probability of surviving from period $n-1$ to period $n$, define ${p}_{n}$ as the post-treatment probability of surviving from period $n-1$ to period $n$, define ${\Pi }_{n}$ as the cumulative post-treatment probability of surviving from period zero to period $n$.^20^ Defining $\beta$ as the one-period discount factor and recalling ${\delta }_{n}\equiv \frac{{\rho }_{n}{H}_{0}}{{\omega }_{H}R}$, the net monetary benefit in the dynamic setting is:
  33 $${\mathrm{NMB}}_{0}=\sum_{n=1}^{N}{\beta }^{n}{\Pi }_{n-1}\left\{[\frac{K{\omega }_{H}{R}_{n}}{{H}_{0}\left(1-{d}^{*}\psi \right)}]\left[{\mu }_{pn}{\delta }_{n}+\phi {p}_{n}{\mu }_{Bn}{\epsilon }_{n}\right]-{p}_{n}\Delta {C}_{n}\right\}-{\Delta C}_{0}.$$
  This expresses net monetary benefit discounted in terms of period zero consumption, $NM{B}_{0}$.
  A suitable incremental generalized risk-adjusted cost-effectiveness ratio (IGRACER) decision rule can be calculated as:
  34 $$\frac{\Delta {C}_{0}+\sum_{n=1}^{N}{\beta }^{n}{\Pi }_{n-1}\left\{{p}_{n}\Delta {C}_{n}\right\}}{\sum_{n=1}^{N}\left\{{\beta }^{n}{\Pi }_{n-1}\left(\frac{{R}_{n}}{{R}_{1}}\right)\left[{\mu }_{pn}{\delta }_{n}+\phi {p}_{n}{\mu }_{Bn}{\epsilon }_{n}\right]\right\}}\le \frac{K{R}_{1}{\omega }_{H}}{{H}_{0}\left(1-\psi{d}^{*} \right)}.$$
  Note that analysts can freely choose any period $j=1,\dots ,N$ to normalize the WTP threshold on the right-hand side, resulting in this more general expression:
  35 $$\frac{\Delta {C}_{0}+\sum_{n=1}^{N}{\beta }^{n}{\Pi }_{n-1}\left\{{p}_{n}\Delta {C}_{n}\right\}}{\sum_{n=1}^{N}\left\{{\beta }^{n }{\Pi }_{n-1}\left(\frac{{R}_{n}}{{R}_{j}}\right)\left[{\mu }_{pn}{\delta }_{n}+\phi {p}_{n}{\mu }_{Bn}{\epsilon }_{n}\right]\right\}}\le \frac{K{R}_{j}{\omega }_{H}}{{H}_{0}\left(1-\psi{d}^{*} \right)}.$$
  As daunting as this might appear, it is simply bookkeeping to summarize results when relevant parameters in the NMB formula vary across multiple periods. In words, where $\text{RASA-WTP}$ represents the risk-adjusted and severity-adjusted WTP, Eqs. (34, 35) simply state that:
  36 $$\frac{\text{Discounted incremental costs}}{\text{Discounted generalized risk-adjusted QALY gains}}\le \mathrm{RASA}\text{-}\mathrm{WTP}.$$
  Equations (34, 35) simplify when treatments have stationary distributions of QoL outcomes (the same in every period). In other words, assume that all parameters in Sect. “Guide to using the GRACE method”B remain constant after treatment is administered. Since the survival probabilities are also constant, ${\mu }_{P}={\mu }_{pn},$ ${p}_{n}=p$, and ${\Pi }_{n}={p}^{n}$, $\forall n\ge 1$. All other parameters are estimated as explained in Sect. “Guide to using the GRACE method” A, B.
  In this case, the *ex ante* net monetary benefit in terms of period zero dollars is:
  37 $$\mathrm{NMB}{}_{\mathrm{exante}}=\left\{\upbeta \left(\frac{1-{(\beta p)}^{N}}{1-\beta p}\right)\right\}\left\{[\frac{K{\omega }_{H}R}{{H}_{0}\left(1-\psi{d}^{*} \right)}]\left[{\mu }_{p}\delta +\phi p{\mu }_{B}\epsilon \right]\right\}-\left\{\Delta {C}_{0}+\sum_{n=1}^{N}{\left(\beta p\right)}^{n}\Delta {C}_{n}\right\}.$$
  The first term in curly braces is the discounted N-period time horizon. The second “curly braces” expression is the risk-, severity-, and disability-adjusted value of per-period improvement in life expectancy and QoL, over the relevant time horizon. The final terms measure the discounted cost of treatment over the N-period time horizon.
  Turning from net monetary benefit to the corresponding IGRACER, technologies with stationary QoL distributions improve *ex ante* welfare if:
  38 $$\frac{\Delta {C}_{0}+\sum_{n=1}^{N}{(\beta p)}^{n}\left\{\Delta {C}_{n}\right\}}{\left[{\mu }_{p}\delta +\phi p{\mu }_{B}\epsilon \right]\beta \left(\frac{1-{(\beta p)}^{N}}{1-\beta p}\right)}\le \frac{K{\omega }_{H}R}{{\mathrm{H}}_{0}\left(1-\psi{d}^{*} \right)}.$$
  which, in words, is identical to Eq. (36).
D. Summary
  Applying GRACE requires new estimates of attitudes towards risk in health outcomes (${r}_{H}^{*}\text{ and }{\pi }_{H}^{*}$), estimation of the rate at which marginal utility in health declines as $H$ increases (${\omega }_{C})$, and estimates of untreated health loss $({\ell}^{*})$ associated with specific individual illnesses. These are “done once” parameters that HTA practitioners themselves will not need to develop; they will appear in the health economics and outcomes research literature, estimated by analysts accomplished at such tasks.

Practitioners assessing new medical technologies must accumulate new statistical evidence, including not only mean differences between new treatments and their comparator therapies, but also differences in variance and skewness between treatments and comparators. They must also estimate mean health losses t* for treated patients, treatment-specific parameters, and measures of uncertainty around those means. GRACE can be applied to static and dynamic contexts, making it suitable for use in Monte Carlo simulations of long-term treatment effects and other similar scenarios. Table 2 summarizes the parameters needed for calculated IGRACERs, along with our proposed estimation methods for each.

**Table 2:** Summary of GRACE parameters to be estimated

Parameter		Fixed (F), or varying over time (T)	Global (G) or treatment-specific (T)	Estimation method
IGRACER components				
Conventional WTP for QALYs	$K$	F	G	$K=\frac{C}{{\omega }_{C}}$
Elasticity of utility with respect to health	${\omega }_{H}$	F	G	Happiness or DCE, as explained in III.D
Severity ratio	$R$	V	T	Derive from Eq. (27)
^†^% QoL loss from permanent disability	${d}^{*}$	F	T	Outcomes studies
^†^Disability ratio	$\psi$	F	T	Derive from Appendix equation (45)
One-period discount rate	$\beta$	F	G	Economic literature
Post-treatment survival probability	$p$	V	T	Outcomes studies
Average increase in survival probability	${\mu }_{p}$	V	T	Outcomes studies
Incremental cost of intervention	$\Delta C$	V	T	Outcomes studies
Marginal rate of substitution between LE and QoL	$\delta$	V	T	$\delta =\frac{\rho {H}_{0}}{{\omega }_{H}R}$; derive $\rho$ from Eqs. (10–12)
Population incidence of disease	$\phi$	F	T	Outcomes studies
Average incremental gain in QoL	${\mu }_{B}$	V	T	Outcomes studies
Certainty–equivalence ratio	$\epsilon$	V	T	Derive using Eq. (32)
Underlying preference parameters				
Annual consumption or income	$C$	F	G	Economic literature
Elasticity of utility with respect to consumption	${\omega }_{C}$	F	G	Economic literature [11, 14, 15]
Relative risk aversion over QoL	${r}_{H}^{*}$	F	G	DCE methods; or happiness methods and Eqs. (24, 25)
Relative prudence over QoL	${\pi }_{H}^{*}$	F	G	DCE methods; or happiness methods and Appendix Eq. (69)
^†^Relative temperance over QoL	${\tau }_{H}^{*}$	F	G	DCE methods; or happiness methods and Appendix Eq. (70)
Underlying outcomes parameters				
Change in variance of QoL outcomes	${\Delta \sigma }_{H}^{2}$	V	T	Outcomes studies
Change in skewness of QoL outcomes	$\Delta \left[{\gamma }_{1}{\sigma }_{H}^{3}\right]$	V	T	Outcomes studies
^†^Change in kurtosis of QoL outcomes	$\Delta {\kappa_H}$	V	T	Outcomes studies
%QoL loss from untreated disease	${\ell}^{*}$	V	T	Outcomes studies
%QoL loss from treated disease	${t}^{*}$	V	T	Outcomes studies
^†^Baseline QoL in excellent health	${H}_{0}$	V	T	Assume ${H}_{0}=1$

^†^Indicates an optional parameter

As with any value assessment model based on utility-maximization of representative individuals, GRACE cannot fully illuminate concerns about inequities of various kinds, unmeasured externalities like scientific spillovers, or macroeconomic effects arising from major contagious pandemics. However, combined with a Rawlsian “veil of ignorance” philosophy [22], the GRACE approach might shed light on some of these issues. GRACE highlights the importance (to utility-maximizing individuals) of valuing health improvements more when health losses are greater, either from acute disease or disability. GRACE also highlights how income or baseline health status might affect value assessments, because ${\omega }_{C}, {\omega }_{H}$, and relative risk preferences may vary with income. Combining these insights about “representative individuals” with a “veil of ignorance” philosophy might help guide public policy on these issues. We leave this for others to pursue.

## Conclusion

GRACE better aligns value assessment with the preferences of real human consumers. Yet, several complexities in the original development of GRACE could impede its adoption and implementation. This paper provides new analyses that (a) generalize the original GRACE formulation to accommodate permanent disability in the baseline period, (b) generalize the original formulation of GRACE to encompass wider ranges of risk preferences, (c) simplify estimation of necessary parameters, (d) outline tractable methods to estimate necessary parameters to implement GRACE models, and (e) extend GRACE to multi-period contexts. We hope that these refinements, combined with the “guidebook” in Sect. “Guide to using the GRACE method”, will help propel forward the implementation of GRACE as the standard HTA model.

The generalization that incorporates disability into the overall WTP value has key public policy importance. The Affordable Care Act prohibits Medicare from using any CEA methods that discriminate against disabled people in making coverage determinations, and similarly prohibits the Patient Centered Outcome Research Institute (PCORI) from using discriminatory CEA methods to measure the value of various medical interventions. The newly refined GRACE model demonstrates that life-extension can be equally or even more valuable to people with disabilities, potentially enabling the use of cost-effectiveness analysis even under current US law.

Finally, our analysis suggests the need for future research on inefficiencies and inequities created by traditional CEA for health insurance programs. Health insurance involves an exchange between consumption today—in the form of premiums paid to private insurers or taxes paid to public insurers—and access to medical technology when sick. In the real-world, health insurance premia and taxes are paid by people of varying health status, often pooled into the same health insurance group. If people with disabilities represent the minority, the presence of healthy consumers serve as a negative externality, inefficiently reducing the generosity of insurance coverage. To see why, imagine two societies, one in which all insurance participants are perfectly healthy *ex ante* (${d}^{*}=0$) and one in which they are all disabled *ex ante* $({d}^{*}>0$). Our analysis implies that optimal insurance coverage could be more generous in the society of the disabled and conversely; this is always true for QoL-improving technologies and may always be true for LE-improving technologies. Since traditional CEA sets coverage decisions as if ${d}^{*}=0$, the resulting coverage may be inefficiently low for disabled people forced to pool with many healthier peers. It thus comes as no surprise that people with disabilities often vigorously oppose the use of traditional CEA to set coverage decisions. Future research should investigate the efficiency rationale for alternative policy solutions, perhaps including the relatively common practice of providing subsidized supplemental coverage for people with disabilities.

As a related matter, future research should estimate how disability affects QoL and QoL utility in healthy and sick states. If disability reduces utility proportionally more (or the same) in healthy states, GRACE implies that disabled people have similar or greater WTP for LE gains. If borne out in the data, this would reinforce GRACE’s existing implication of higher WTP for QoL gains among the disabled. GRACE provides a potential path forward for satisfying the requirements of the Affordable Care Act regarding disability-related discrimination, thus allowing use of CEA both in PCORI evaluations and in setting coverage determinations in Medicare.

## Appendix I: extensions of GRACE

A. Derivation of disability-adjusted WTP
  In the text, we claim that $\frac{W\left({H}_{0d}\right)}{W\left({H}_{0}\right)}=1-{d}^{*}\psi$, where $\psi$ depends on ${\omega }_{H}$, relative risk-aversion over QoL (${r}_{H}^{*}$), and relative prudence over QoL (${\pi }_{H}^{*}$). We now define $\psi$ more specifically and prove this claim. Begin by computing a Taylor Series expansion of $W({H}_{0d})$ around $W({H}_{0})$:
  39 $${W(H_{{0d}} )} \approx W\left( {H_{0} } \right) + W^{\prime}\left( {H_{0} } \right)\left( {H_{{0d}} - H_{0} } \right) + \frac{1}{2}W^{\prime\prime}\left( {H_{0} } \right)\left( {H_{{0d}} - H_{0} } \right)^{2} + \frac{1}{6}W^{\prime\prime\prime}\left( {H_{0} } \right)\left( {H_{{0d}} - H_{0} } \right)^{3} + \dots.$$
  We divide (39) by $W\left({H}_{0}\right)$ to recover an expression for $\frac{W\left({H}_{0d}\right)}{W\left({H}_{0}\right)}$, and we then analyze the resulting expression term by term:
  40 $$First\; Term: \frac{W\left({H}_{0}\right)}{W\left({H}_{0}\right)}=1.$$
  The second term is $\frac{{W}^{^{\prime}}\left({H}_{0}\right)({H}_{0d}-{H}_{0})}{W\left({H}_{0}\right)}$. Multiply and divide this term by ${H}_{0}$, recognizing that the component parts are ${\omega }_{H}$ and ${d}^{*}\equiv \frac{\left({H}_{0}-{H}_{0d}\right)}{{H}_{0}},$ the expected relative loss in QoL due to permanent disability. With these definitions:
  41 $$Second\; Term: -{\omega }_{H}{d}^{*}.$$
  The third term is $\frac{1}{2}\frac{{W^{\prime\prime}\left( {{\text{H}}_{{\text{0}}} } \right)\left( {{\text{H}}_{{{\text{0d}}}} - {\text{H}}_{{\text{0}}} } \right)^{{\text{2}}} }}{{{\text{W}}\left( {{\text{H}}_{{\text{0}}} } \right)}}$. Now multiply and divide by ${W}^{^{\prime}}\left({H}_{0}\right).$ Since absolute risk aversion is $r_{H} \equiv - \frac{{W^{\prime\prime}\left( {{\text{H}}_{{\text{0}}} } \right)}}{{W^{\prime}\left( {{\text{H}}_{{\text{0}}} } \right)}},$ this becomes $-\frac{1}{2}\left\{{r}_{H}\right\}\frac{\left\{E{\left({H}_{0d}- {H}_{0}\right)}^{2}\right\}{W}^{^{\prime}}\left({H}_{0}\right)}{W\left({H}_{0}\right)}.$ Now multiply and divide by ${H}_{0}^{2}$, allocating one ${H}_{0}$ term in the numerator to convert absolute risk aversion, *r*, into relative risk aversion, ${r}_{H}^{*}$, and the other to complete ${\omega }_{H}$:
  42 $$Third\; Term: -\frac{1}{2}{\omega }_{H}{r}_{H}^{*}{{d}^{*}}^{2}.$$
  Note that ${r}_{H}^{*}$ is defined at ${H}_{0}$, whereas, in [1], it is defined at the average level of untreated health, ${\mu }_{H}$. Therefore, analysts must either assume that ${r}_{H}^{*}$ is constant across all levels of health (CRRA) or they must estimate ${r}_{H}^{*}$ at baseline health level ${H}_{0}$ as well as at ${H}_{1S}$.
  The fourth term of (39) is $\frac{1}{6}W^{\prime\prime\prime}\left( {{\text{H}}_{{\text{0}}} } \right)\left( {{\text{H}}_{{{\text{0d}}}} - {\text{H}}_{{\text{0}}} } \right)^{{\text{3}}}$. Dividing by $W\left({H}_{0}\right)$, gives $\frac{1}{6}[W^{\prime\prime\prime}\left( {{\text{H}}_{{\text{0}}} } \right)\left( {{\text{H}}_{{{\text{0d}}}} - {\text{H}}_{{\text{0}}} } \right)^{{\text{3}}} ]\frac{{\text{1}}}{{{\text{W}}\left( {{\text{H}}_{{\text{0}}} } \right)}}$. Defining absolute prudence as $\pi _{H} \equiv \frac{{ - W^{\prime\prime\prime}\left( {{\text{H}}_{{\text{0}}} } \right)}}{{W^{\prime\prime}\left( {{\text{H}}_{{\text{0}}} } \right)}},$ multiplying and dividing by $W^{\prime\prime}\left( {{\text{H}}_{{\text{0}}} } \right)$ gives $- \frac{1}{6}\pi _{H} [W^{\prime\prime}\left( {{\text{H}}_{{\text{0}}} } \right)]\left( {{\text{H}}_{{{\text{0d}}}} - {\text{H}}_{{\text{0}}} } \right)^{{\text{3}}} ]\frac{{\text{1}}}{{{\text{W}}\left( {{\text{H}}_{{\text{0}}} } \right)}}$. Multiplying and dividing by ${W}^{^{\prime}}\left({H}_{0}\right)$, gives $\frac{1}{6}{{\pi }_{H}{r}_{H}}{\left({H}_{0d}- {H}_{0}\right)}^{3}]\frac{{W}^{^{\prime}}\left({H}_{0}\right)}{W\left({H}_{0}\right)}$. Next, multiply and divide by ${H}_{0}^{3}$, allocating two of the ${H}_{0}$ terms in the numerator to the risk-valuation terms and the other to complete the definition of ${\omega }_{H}$, Then:
  43 $$Fourth\; Term: -\frac{1}{6}{\pi }_{H}^{*}{r}_{H}^{*}{\omega }_{H}{{d}^{*}}^{3}.$$
  As with relative risk-aversion, analysts must either assume that ${\pi }_{H}^{*}$ is constant across all levels of health (CRRA) or they must estimate ${\pi }_{H}^{*}$ at baseline health level ${H}_{0}$ as well as at ${H}_{1S}$. Any fifth term involving kurtosis would follow the same general strategy.
  In summation, the Taylor Series approximation to $\frac{W({H}_{0d})}{W\left({H}_{o}\right)}$ is:
  44 $$\frac{W\left({H}_{0d}\right)}{W\left({H}_{0}\right)}=\left[1-{d}^{*}({\omega }_{H}+\frac{1}{2}{\omega }_{H}{r}_{H}^{*}{d}^{*}+\frac{1}{6}{\omega }_{H}{r}_{H}^{*}{\pi }_{H}^{*}{\left({d}^{*}\right)}^{2}+\dots )\right].$$
  Finally, defining:
  45 $$\psi \equiv {\omega }_{H}\left(1+\frac{1}{2}{r}_{H}^{*}{d}^{*}+\frac{1}{6}{r}_{H}^{*}{\pi }_{H}^{*}{\left({d}^{*}\right)}^{2}+\dots \right),$$
  completes the proof that $\frac{W\left({H}_{0d}\right)}{W\left({H}_{0}\right)}=1-{d}^{*}\psi$.
  Inspection of (45) shows that $\psi >0$, since ${\omega }_{H}>0$ and all other components are weakly positive. Next, we show that $\psi \le 1$ under weak risk-aversion. Exploiting the definition of ${d}^{*}$, we can rearrange $\frac{W\left({H}_{0d}\right)}{W\left({H}_{0}\right)}=1-{d}^{*}\psi$ to show that:
  46 $$\psi =\frac{\frac{\left(W\left({H}_{0}\right)-W\left({H}_{0d}\right)\right)}{\left({H}_{0}-{H}_{0d}\right)}}{\frac{W\left({H}_{0}\right)-W(0)}{{H}_{0}-0}}.$$
  Here, we implicitly assume that $W\left(0\right)=0$. This equation expresses $\psi$ as the ratio of two slopes: (1) The average slope of $W$ between ${H}_{0d}>0$ and ${H}_{0}$; and (2) the average slope of $W$ between $0$ and ${H}_{0}$. For a strictly concave, increasing utility function, $W$, the slope between ${H}_{0d}$ and ${H}_{0}$ will be strictly less than the slope between $0$ and ${H}_{0}$. For a linear utility function, on the other hand, the slopes will be equal. This proves the necessary result.
B. Proof of the reformulation of ${\varvec{\delta}}$
  Define $V\equiv E\left[\phi W\left({H}_{1S}+B\right)\right]+\left(1-\phi \right)W\left({H}_{1W}\right)$, the expected utility in period 1, conditional on survival to that period. In LP [1], the marginal rate of substitution is defined as $\delta =\frac{V}{{W}^{^{\prime}}\left({\mu }_{H}\right)}$, the ratio of marginal utility of increased LE to marginal utility of QoL in period 1. In [1], it was necessary to estimate this for each illness/treatment combination.
  We begin by proving the equivalence of Eqs. (1) and (9) in the main text. By inspection, we can see that this equivalence rests on the proposition that ${\omega }_{H}R\delta =\rho {H}_{0}$. To prove the latter, note that LP define $\delta \equiv [\frac{V}{{W}^{^{\prime}}\left({\mu }_{H}\right)}]$, $R\equiv [\frac{{W}^{^{\prime}}\left({\mu }_{H}\right)}{{W}^{^{\prime}}\left({H}_{0}\right)}]$, and ${\omega }_{H}\equiv [\frac{{W}^{^{\prime}}\left({H}_{0}\right){H}_{0}}{W\left({H}_{0}\right)}]$. Therefore, ${\omega }_{H}R\delta =\left[\frac{{W}^{^{\prime}}\left({H}_{0}\right){H}_{0}}{W\left({H}_{0}\right)}\right]\left[\frac{{W}^{^{\prime}}\left({\mu }_{H}\right)}{{W}^{^{\prime}}\left({H}_{0}\right)}\right][\frac{V}{{W}^{^{\prime}}\left({\mu }_{H}\right)}]=\frac{V}{W\left({H}_{0}\right)}{H}_{0}$. Since $\rho \equiv \frac{V}{W\left({H}_{0}\right)}$, the result follows, and Eqs. (1) and (9) are equivalent.
  Next, for compactness, define ${H}_{T}\equiv \left({H}_{1S}+B\right)$, where T stands for “treated” health outcome. Next, define ${V}_{S}\equiv W\left({H}_{T}\right),$ with a corresponding value of ${\rho }_{S}=\frac{{V}_{s}}{W({H}_{o})}$. In parallel, define ${V}_{H}=W\left({H}_{1W}\right)$ and ${\rho }_{W}=\frac{{V}_{W}}{W({H}_{0})}.$ This allows us to think of $V$ as the probability-weighted average of period 1 utility in the sick state and period 1 utility in the well state. It also allows us to rewrite $\rho$ as $\rho =\phi {\rho }_{S}+\left(1-\phi \right){\rho }_{W}.$
  To estimate ${\rho }_{S}$, consider the Taylor series expansion of ${V}_{S}$ around ${H}_{0}$, which proceeds quite similarly to the expansion of $W({H}_{0d})$ around $W({H}_{0})$ above:
  47 $$E\left( {V_{S} } \right) \approx W\left( {H_{0} } \right) + W^{\prime}\left( {H_{0} } \right)E\left( {H_{T} - H_{0} } \right) + \frac{1}{2}W^{\prime\prime}\left( {H_{0} } \right)E\left( {H_{T} - H_{0} } \right)^{2} + \frac{1}{6}W^{\prime\prime\prime}\left( {H_{0} } \right)E\left( {H_{T} - H_{0} } \right)^{3} + \ldots .$$
  Divide (47) by $W\left({H}_{0}\right)$, following the definition of ${\rho }_{S}$, and analyze it term by term:
  48 $$First\; Term: \frac{W\left({H}_{0}\right)}{W\left({H}_{0}\right)}=1.$$
  The second term is $\frac{{W}^{^{\prime}}\left({H}_{0}\right)E\left({H}_{T}-{H}_{0}\right)}{W\left({H}_{0}\right)}$. Multiply and divide this term by ${H}_{0}$, recognizing that the component parts are ${\omega }_{H}$ (defined at ${H}_{0}$) and ${t}^{*}\equiv \frac{{H}_{0}-E\left({H}_{T}\right)}{{H}_{0}},$ the expected relative loss in health status after treatment. With these definitions:
  49 $$Second\; Term: -{\omega }_{H}{t}^{*}.$$
  The third term is $\frac{1}{2}\frac{{W^{\prime\prime}\left( {{\text{H}}_{{\text{0}}} } \right){\text{E}}\left( {{\text{H}}_{{\text{T}}} - {\text{H}}_{{\text{0}}} } \right)^{{\text{2}}} }}{{{\text{W}}\left( {{\text{H}}_{{\text{0}}} } \right)}}$. Multiply and divide by $W^{\prime}\left( {H_{0} } \right)H_{0}^{2} ,$ allocating one ${H}_{0}$ term to the numerator to convert absolute risk aversion, *r*, into relative risk aversion, ${r}_{H}^{*}$, and the other to complete ${\omega }_{H}$. With these algebraic changes:
  50 $$Third\; Term: -\frac{1}{2}{\omega }_{H}{r}_{H}^{*}{t}^{*2}.$$
  As above in Appendix IA, ${r}_{H}^{*}$ is measured at ${H}_{0}$.
  The fourth term of (47) is $\frac{1}{6}W^{\prime\prime\prime}\left( {{\text{H}}_{{\text{0}}} } \right){\text{E}}\left( {{\text{H}}_{{\text{T}}} - {\text{H}}_{{\text{0}}} } \right)^{{\text{3}}}$. Dividing by $W\left({H}_{0}\right)$, gives $\frac{1}{6}[W^{\prime\prime\prime}\left( {{\text{H}}_{{\text{0}}} } \right){\text{E}}\left( {{\text{H}}_{{\text{T}}} - {\text{H}}_{{\text{0}}} } \right)^{{\text{3}}} ]\frac{{\text{1}}}{{{\text{W}}\left( {{\text{H}}_{{\text{0}}} } \right)}}$. Multiply and divide by $W^{\prime}\left( {H_{0} } \right)W^{\prime\prime}({\text{H}}_{{\text{0}}} ){\text{H}}_{{\text{0}}}^{{\text{3}}}$. Then:
  51 $$Fourth\; Term: -\frac{1}{6}{\pi }_{H}^{*}{r}_{H}^{*}{\omega }_{H}{t}^{*3}.$$
  As before, ${\pi }_{H}^{*}$ is defined at ${H}_{0}$. Any fifth term involving kurtosis would follow the same general strategy.
  In summation, the Taylor Series approximation to $\frac{{V}_{S}}{W\left({H}_{o}\right)}\equiv {\rho }_{S}$ is:
  52 $${\rho }_{S}\approx 1-{\omega }_{H}t^*[1+\frac{1}{2}{r}_{H}^{*}{t}^{*}+\frac{1}{6}{{\pi }_{H}^{*}{r}_{H}^{*}{t}^{*2}}^{ }+\dots ].$$
  Turning to the remaining portion of $V$, we presume that ${H}_{1W}={H}_{0d}$ in the sense that permanent disability (if present) persists across periods. As in the main text, define ${d}^{*}\equiv \frac{{H}_{0}-{H}_{0d}}{{H}_{0}}=\frac{{H}_{0}-{H}_{1W}}{{H}_{0}}$, the relative QoL loss from permanent disability in either period zero or period one. This allows us to write the parallel Taylor expansion:
  53 $${\rho }_{W}=1-{\omega }_{H}d^*\left[1+{\frac{1}{2}r_H^*}{d^*}+{\frac{1}{6}{\pi }_{H}^{*}r_{H}^*}{d}^{{*}^{2}}+\dots \right].$$
  Then overall:
  54 $$\rho =\phi {\rho }_{S}+\left(1-\phi \right){\rho }_{W}.$$
C. Proof of results relating to disability-adjusted WTP

First, we show that permanent disability increases the WTP for improvements in average QoL. Equation (7) implies that the marginal value of an improvement in average QoL is given by $\frac{K{\omega }_{H}R}{{H}_{0}(1-\psi{d}^{*} )}\phi {p}_{1}\epsilon$. Disability increases $\frac{1}{1-\psi{d}^{*} }$. Moreover, if utility exhibits CRRA over QoL, all other terms are unaffected by disability. Technically, disability could have complex effects on ${\omega }_{H}$, $\psi$, and $\epsilon$, if relative risk-aversion varies with QoL. In this case, the relationship between disability and QoL needs to be judged empirically.

Turning to life-extension, Eq. (1) implies that the value of life-extension, ${\mu }_{p}$, is given by $K{\omega }_{H}R{\mu }_{p}\delta .$ The total disability-adjusted value of life-extension is thus given by $TDVLE\equiv \frac{K{\omega }_{H}R{\mu }_{p}\delta }{{H}_{0}(1-\psi{d}^{*} )}$.

Appendix IB showed that $\delta =\frac{\rho {H}_{0}}{{\omega }_{H}R}$. Using the definition of $\rho$, this becomes $\delta =\frac{V{H}_{0}}{W({H}_{0}){\omega }_{H}R}$. Moreover, by definition, $\frac{W\left({H}_{0d}\right)}{W\left({H}_{0}\right)}=1-\psi{d}^{*}$. Substituting these two terms into $TDVLE$ yields:

55 $$TDVLE\equiv K{\mu }_{p}\frac{V}{W\left({H}_{0d}\right)}$$

Exploiting the definition of $V\equiv E\left[\phi W\left({H}_{1S}+B\right)\right]+\left(1-\phi \right)W\left({H}_{1W}\right)$ from Appendix IB, this becomes:

56 $$TDVLE\equiv K{\mu }_{p}\left[\phi \frac{E\left[W\left({H}_{1S}+B\right)\right]}{W\left({H}_{0d}\right)}+\left(1-\phi \right)\right].$$

If permanent disability weakly increases $\frac{E\left(W\left({H}_{1S}+B\right)\right)}{W\left({H}_{0d}\right)}$, $TDVLE$ weakly rises (holding ${\mu }_{p}$ fixed), and vice-versa.

Next, we show for illustrative purposes that people with disabilities have equal WTP for LE under CRRA utility and multiplicative disability. To focus on the disability effects, assume that illness and treatment effects are non-stochastic. Assume further that utility belongs to the HARA family. We implement multiplicative disability by assuming that ${H}_{0d}={H}_{0}(1-{d}^{*})$ and ${H}_{1S}+B={H}_{0}(1-{i}_{t}^{*})(1-{d}^{*})$, where $0<{i}_{t}^{*}<1$ represents the percentage loss in QoL from acute treated illness and ${d}^{*}$ continues to represent the percentage loss in QoL from disability. Define the functions ${\omega }_{H}\left({H}_{0d}\right)\equiv \frac{{W}^{^{\prime}}\left({H}_{0d}\right){H}_{0d}}{W\left({H}_{0d}\right)}$ and ${\omega }_{H}\left({H}_{1S}+B\right)\equiv \frac{{W}^{^{\prime}}\left({H}_{1S}+B\right)\left({H}_{1S}+B\right)}{W\left({H}_{1S}+B\right)}$, the elasticities of utility with respect to QoL evaluated as indicated. Differentiation implies that:

57 $$\frac{\partial }{\partial {d}^{*}}\left[\frac{W\left({H}_{1S}+B\right)}{W\left({H}_{0d}\right)}\right]=\frac{\left[{W}^{^{\prime}}\left({H}_{0d}\right){H}_{0}W\left({H}_{1S}+B\right)-{W}^{^{\prime}}\left({H}_{1S}+B\right){H}_{0}\left(1-{i}_{t}^{*}\right)W\left({H}_{0d}\right)\right]}{W{\left({H}_{0d}\right)}^{2}}.$$

This can be simplified as:

58 $$\frac{\partial }{\partial {d}^{*}}\left[\frac{W\left({H}_{1S}+B\right)}{W\left({H}_{0d}\right)}\right]=W\left({H}_{0d}\right)W\left({H}_{1S}+B\right)\frac{\left[\frac{{W}^{^{\prime}}\left({H}_{0d}\right){H}_{0d}}{W\left({H}_{0d}\right)} \frac{{H}_{0}}{{H}_{0d}}-\frac{{W}^{^{\prime}}\left({H}_{1S}+B\right)\left({H}_{1S}+B\right)}{W\left({H}_{1S}+B\right)} \frac{{H}_{0}\left(1-{i}_{t}^{*}\right)}{{H}_{1S}+B}\right]}{W{\left({H}_{0d}\right)}^{2}}.$$

Observe that, by definition, $\frac{{H}_{0}}{{H}_{0d}}=\frac{1}{1-{d}^{*}}=\frac{{H}_{0}\left(1-{t}^{*}\right)}{{H}_{1S}+B}$. And, when utility is CRRA, Eq. (16) implies that the elasticity of utility is constant, so that $\frac{{W}^{^{\prime}}\left({H}_{0d}\right){H}_{0d}}{W\left({H}_{0d}\right)}=\frac{{W}^{^{\prime}}\left({H}_{1S}+B\right)\left({H}_{1S}+B\right)}{W\left({H}_{1S}+B\right)}$. It thus follows that $\frac{\partial }{\partial {d}^{*}}\left[\frac{W\left({H}_{1S}+B\right)}{W\left({H}_{0d}\right)}\right]=0$.

Finally, we derive sufficient conditions under which permanent disability increases $\frac{E\left(W\left({H}_{1S}+B\right)\right)}{W\left({H}_{0d}\right)}$. Consider the Taylor Series expansion of $E(W\left({H}_{1S}+B\right))$ around ${H}_{0d}$. Following the same approach used in Appendix IA and Appendix IB, one can show:

59 $$\frac{E\left(W\left({H}_{1S}+B\right)\right)}{W\left({H}_{0d}\right)}=1-{\omega }_{H}\left(\frac{{t}^{*}-{d}^{*}}{1-{d}^{*}}\right)-{\omega }_{H}{r}_{H}^{*}{\left(\frac{{t}^{*}-{d}^{*}}{1-{d}^{*}}\right)}^{2}-{\omega }_{H}{r}_{H}^{*}{\pi }_{H}^{*}{\left(\frac{{t}^{*}-{d}^{*}}{1-{d}^{*}}\right)}^{3}-\dots .$$

Note that in this case, the relative risk preference terms are evaluated at ${H}_{0d}$. Under CRRA preferences, the relative risk preference terms do not vary with ${d}^{*}$. In this case, it is clear that $\frac{\partial }{\partial {d}^{*}}\frac{E\left(W\left({H}_{1S}+B\right)\right)}{W\left({H}_{0d}\right)}\ge 0$ if and only if $\frac{\partial }{\partial {d}^{*}}\left(\frac{{t}^{*}-{d}^{*}}{1-{d}^{*}}\right)\le 0$.

60 $$\frac{\partial }{\partial {d}^{*}}\left(\frac{{t}^{*}-{d}^{*}}{1-{d}^{*}}\right)=\frac{\left(\frac{\partial {t}^{*}}{\partial {d}^{*}}-1\right)\left(1-{d}^{*}\right)+\left({t}^{*}-{d}^{*}\right)}{{\left(1-{d}^{*}\right)}^{2}}.$$

This derivative will be weakly negative if and only if:

61 $$\frac{\partial {t}^{*}}{\partial {d}^{*}}\le \frac{1-{t}^{*}}{1-{d}^{*}}\le 1.$$

Expression (61) requires that disability lowers QoL in the sick state by less than in the healthy state. For curative therapies, we require $\frac{\partial {t}^{*}}{\partial {d}^{*}}\le 1$, which is always true, but for less than perfect therapies, the requirement becomes non-trivial.

A useful alternate formulation of this expression arises from the change of variables, $D\equiv 1-{d}^{*}$ and $T\equiv 1-{t}^{*}$. $D$ and $T$ represent the QoL index in the healthy and treated sick states, respectively, in the presence of disability. Notice that $\left(\frac{{t}^{*}-{d}^{*}}{1-{d}^{*}}\right)=\left(\frac{D-T}{D}\right)=1-\frac{T}{D}$. Moreover, $\frac{\partial }{\partial D}\left(\frac{D-T}{D}\right)=-\frac{\left(\frac{\partial T}{\partial D}D-T\right)}{{D}^{2}}$. Observe that $\frac{\partial }{\partial D}\left(\frac{D-T}{D}\right)\le 0$ if and only if $\left(\frac{\partial T}{\partial D}\frac{D}{T}\right)\ge 1$. In other words, reducing disability so that healthy state QoL rises by one percent must increase treated sick state QoL by more than one percent.

## Appendix II: simplied hyperbolic absolute risk-aversion (HARA) functions

Traditional HARA utility functions are defined as:

62 $$U\left(H\right)=\frac{1-\gamma }{\gamma }{Z}^{\gamma },\mathrm{ where }Z=\frac{aH}{1-\gamma }+\eta .$$

Since H is defined on the [0,1] domain, arbitrary rescaling using the parameter $a$ provides no additional generality. Therefore, a convenient simplification defines $a\equiv \left(1-\gamma \right).$ Then, since $Z=H+\eta$, we can write:

63 $$U^{\prime}\left( H \right) = \left( {1 - \gamma } \right)Z^{{\gamma - 1}} ,$$

64 $$U^{\prime\prime}\left( H \right) = \left( {\gamma - 1} \right)\left( {1 - \gamma } \right)Z^{{\gamma - 2}} ,$$

from which we have ${r}_{H}=\frac{1-\gamma }{Z} \text{ and therefore:}$

65 $${r}_{H}^{*}=\left(1-\gamma \right)\left(\frac{H}{Z}\right).$$

Similarly:

66 $$\omega _{H} = \frac{{U^{\prime}\left( H \right)H}}{{U\left( H \right)}}= \gamma \left( {\frac{H}{Z}} \right)$$

Importantly, since the $\left(\frac{H}{Z}\right)$ values cancel out, dividing (65) by (66) yields:

67 $$\frac{{\mathrm{r}}_{\mathrm{H}}^{*}}{{\omega }_{H}}=\frac{1-\gamma }{\gamma },$$

from which we have:

68 $$\upgamma =\frac{{\upomega }_{\mathrm{H}}}{{\upomega }_{\mathrm{H}}+{\mathrm{r}}_{\mathrm{H}}^{*}}.$$

Since $U^{\prime\prime\prime}\left( H \right) = (\gamma - 2)\left( {\gamma - 1} \right)\left( {1 - \gamma } \right)Z^{{\gamma - 3}} ,$ similar calculations demonstrate that:

69 $${\pi }_{H}^{*}=\left(2-\gamma \right)\left(\frac{H}{Z}\right)=\frac{\left(2-\upgamma \right)}{\left(1-\gamma \right)}{r}_{H}^{*}.$$

Similarly:

70 $${\tau }_{H}^{*}=\left(3-\gamma \right)\left(\frac{H}{Z}\right)=\left[\frac{3-\gamma }{(1-\gamma )}\right]{r}_{H}^{*}.$$

The general form for the jth relative QoL risk preference parameter, ${\zeta }_{jH}^{*}$ is:

71 $${\zeta }_{j}^{*}=\left[\frac{j-\gamma }{1-\gamma }\right]{r}_{H}^{*}.$$

Moreover, as proven in [15], Eq. (69) allows the recovery of $\gamma$ from ${r}_{H}^{*}$ and ${\pi }_{H}^{*}$ as in:

72 $$\gamma =\frac{{\pi }_{H}^{*}-2{r}_{H}^{*}}{{\pi }_{H}^{*}-{r}_{H}^{*}}.$$

## Appendix III: consequences of non-CRRA utility for estimation of ${\varvec{R}}$

Recall eqs. (69) to (71) for definitions of higher-order risk parameters. In contrast, for CRRA utility, ${\pi }_{H}^{*}=1+{r}_{H}^{*}, {\tau }_{H}^{*}=2+{r}_{H}^{*}, \text{ and }{\zeta }_{j}^{*}=(j-1)+{r}_{H}^{*}$. (Observe that $j=1$ for relative risk-aversion, $j=2$ for relative prudence, and so on.) Now suppose that the true utility function is within the HARA family but does not satisfy CRRA (i.e., $\eta \ne 0)$. Where ${r}_{H}^{*}=1$ and using the above expressions, we can estimate the relative bias that would follow from incorrectly assuming CRRA utility. Now define $\widehat{{\zeta }_{j}^{*}}$ as the CRRA-based estimate and ${\zeta }_{j}^{*}$ as the true value. Therefore:

73 $$Relative\text{ }Bias=\frac{\widehat{{\zeta }_{j}^{*}}}{{\zeta }_{j}^{*}}-1=(1-\gamma )\frac{1+\left[\frac{j-1}{{r}^{*}}\right]}{\left[j-\gamma \right]}-1.$$

With ${r}^{*}=1$, relative bias becomes:

74 $$Relative\text{ }Bia{s|}_{{r}^{*}=1}=\frac{j(1-\gamma )}{j-\gamma }-1.$$

If “relative bias” is 10%, CRRA-based estimates will understate higher-order risk parameters by 10%, etc. Table 3 reports these relative bias figures for various values of $\gamma$ (along the top row) and for relative risk parameters of differing degrees, $j$. As noted above, these estimates refer to the case where ${r}^{*}=1$. The left-hand column covers the case of $\gamma =0$, under which CRRA is the correctly specified utility function (Table 3).

**Table 3:** Relative bias of relative risk preferences when CRRA is incorrectly specified

	$$\gamma$$					
	0.0	0.2	0.4	0.5	0.6	0.8
${\pi }^{*}$	0%	− 11%	− 25%	− 33%	− 43%	− 67%
${\tau }^{*}$	0%	− 14%	− 31%	− 40%	− 50%	− 73%
${\zeta }_{4}^{*}$	0%	− 16%	− 33%	− 43%	− 53%	− 75%
${\zeta }_{5}^{*}$	0%	− 17%	− 35%	− 44%	− 55%	− 76%
${\zeta }_{6}^{*}$	0%	− 17%	− 36%	− 45%	− 56%	− 77%

The larger is $\gamma$, the greater the potential for relative bias when relying on the CRRA assumption. Indeed, if $\gamma \ge 0.5$, the relative bias approaches or exceeds 50%.

Table 3 assumes ${r}_{H}^{*}=1$. Equation (73) implies that larger relative risk-aversion parameters somewhat mitigate the relative bias reported in the table, and vice-versa. Furthermore, all the higher-order terms are moot if the relevant health outcomes have normal distributions, since all statistical moments above variance would then equal zero.

## Appendix IV: relationships between utility parameters and their estimation using happiness regression coefficients

First, consider ${\omega }_{H}=\frac{{W}^{^{\prime}}\left(H\right)H}{W\left(H\right)},$ the elasticity of utility with respect to H. Then, generally:

75 $$\frac{\partial {\omega }_{H}}{\partial H}=\frac{1}{W}\left[{W}^{{{\prime}}}+[\frac{{W}^{{{\prime}}{{\prime}}}}{{W}^{{{\prime}}}}]W{{\prime}}H\right]-\frac{1}{{W}^{2}}\left[H{W}^{{{\prime}}}{W}^{{{\prime}}}\right]=\frac{{W}^{{{\prime}}}}{W}\left[1-{r}_{H}^{*}\right]-\left[\frac{{\omega }_{H}^{2}}{H}\right].$$

From this:

76 $${\epsilon }^{\omega }=1-{r}_{H}^{*}-{\omega }_{H},$$

and thus:

77 $${r}_{H}^{*}=\left(1-{\omega }_{H}\right)-{\epsilon }^{\omega }.$$

Now consider a second-order happiness regression:

78 $$\mathrm{ln}\left(Happy\right)=\;{\beta }_{1}\mathrm{ln}\left(H\right)+\frac{1}{2}{\beta }_{2}{\mathrm{ln}\left(H\right)}^{2}+{\beta }_{3}\mathrm{ln}\left(C\right)+\frac{1}{2}{\beta }_{4}{\mathrm{ln}\left(C\right)}^{2}+{\beta }_{5}\mathrm{ln}\left(H\right)\mathrm{ln}\left(C\right)+\epsilon .$$

Suppressing the interaction term to preserve clarity (i.e., setting ${\beta }_{5}=0$):

79 $$\frac{\partial \mathrm{ln}\left(Happy\right)}{\partial \mathrm{ln}\left(H\right)}\equiv {\omega }_{H}={\beta }_{1}+{\beta }_{2}\mathrm{ln}\left(H\right).$$

This implies the empirical analogs:

80 $$\widehat{{\omega }_{H}}=\widehat{{\beta }_{1}}+\widehat{{\beta }_{2}}\mathrm{ln}\left(H\right),$$

81 $$\widehat{\frac{\partial {\omega }_{H}}{\partial H}}=\frac{\widehat{{\beta }_{2}}}{H}.$$

From this:

82 $$\widehat{{\epsilon }^{\omega }}=\frac{\partial \widehat{{\omega }_{H}}}{\partial H}\left(\frac{H}{\widehat{{\omega }_{H}}}\right)=\frac{\widehat{{\beta }_{2}}}{\widehat{{\omega }_{H}}}.$$

Finally, using eq. (80) and (82), and referring to (77), we can estimate relative risk-aversion as:

83 $$\widehat{{r}_{H}^{*}}=\left(1-\widehat{{\omega }_{H}}\right)-\frac{\widehat{{\beta }_{2}}}{\widehat{{\omega }_{H}}}=1-\left(\widehat{{\beta }_{1}}+\widehat{{\beta }_{2}}\mathrm{ln}\left(H\right)\right)-\frac{\widehat{{\beta }_{2}}}{\left(\widehat{{\beta }_{1}}+\widehat{{\beta }_{2}}\mathrm{ln}\left(H\right)\right)}.$$

Higher-order estimates follow by using (68) to estimate $\gamma$ using estimates of ${\omega }_{H}$ and ${r}_{H}^{*}$, and by using eq. (69) to (71) to compute the higher-order risk preference parameters using estimates of $\gamma$ and ${r}_{H}^{*}$.

## Footnotes

1 Two recent studies assess whether there are diminishing returns to QoL. Attema et al. [4] provide an estimate of the elasticity of utility with respect to health of 0.8. Nielsen et al. [5] estimated the effects of disease severity on willingness to pay (WTP). Across their various specifications, the elasticity of WTP with respect to illness severity ranged from 0.12 to 0.30.

2 Within Box 1, the two terms marked with †, namely ${\pi }_{H}^{*}$ and ${r}_{H}^{*}$, are evaluated at ${H}_{0}$ when used to estimate $\psi$ and $\rho$, but evaluated at ${H}_{1S}$ otherwise.

3 We assume throughout that ${H}_{1W}={H}_{0d}$, so that permanent disability persists from period zero onwards. This generalization also requires that ${H}_{1S}$ include permanent disability; therefore, analysts must calculate ${H}_{1s}$ to reflect the effects of untreated acute illness and any pre-existing permanent disability.

4 As discussed in Appendix IC, these claims rely on constant relative risk-aversion over QoL. If risk preferences instead vary with QoL, a variety of complex effects may arise, and the relationship must be studied empirically.

5 See the discussion around Eq. (8) below.

6 Section 1182, 42 U.S.C. 1320e–1(c(1)) reads: “The Patient-Centered Outcomes Research Institute established under Sect. 1181(b)(1) shall not develop or employ a dollars-per-quality adjusted life year (or similar measure that discounts the value of a life because of an individual’s disability) as a threshold to establish what type of health care is cost effective or recommended. The Secretary shall not utilize such an adjusted life year (or such a similar measure) as a threshold to determine coverage, reimbursement, or incentive programs under title XVIII”.

7 Here and elsewhere, the results for $\delta$ hold under the permanent disability generalization, but we suppress the additional notation for simplicity.

8 In [1, p. 9], due to a typographical error, this was defined as $\ell ^{*} \equiv H_{0} - \mu _{H}$. Box 1 correctly defines $^{*} \equiv \frac{{H_{0} - \mu _{H} }}{{H_{0} }}$, a distinction that matters only in exceptional cases where analysts wish to set ${H}_{0} \ne 1$.

9 For comparison, Nielson et al. [5] estimated that the elasticity of R with respect to illness severity was (at their midpoint) 0.2. This is approximately consistent with the values in Table 1 when ${r}_{H}^{*}=0.25.$

10 See Eqs. (14) and (15) and surrounding discussion for explanation of why ${\omega }_{H}=1-{r}_{H}^{*}$ under CRRA.

11 For an expanded discussion of HARA utility, see [15].

12 When $\eta <0$, this formulation closely resembles the widely-used Stone-Geary utility function, where the $\eta$ parameter represents minimum values of consumption before any utility is produced.

13 In CRRA utility, the higher order risk preferences are determined by ${\pi }^{*}={r}^{*}+1, {\tau }^{*}={r}^{*}+2, \dots . etc.$

14 For normal distributions, the risk terms above variance all equal zero.

15 As noted in Appendix IA and B, the estimation of $\delta$ and $\psi$ require relative risk preference parameters evaluated at ${H}_{0}$.

16 The variance of the estimated Pearson Skewness parameter in normal populations is approximately 6/n for sufficiently large samples.

17 The variance of estimated excess kurtosis in normal samples is approximately 24/n for sufficiently large samples.

18 This is true when ${d}^{*}=0$. When ${d}^{*}>0, { t}^{*}={d}^{*}.$

19 While we suppress the extra notation, note that variance and skewness in both the treatment and comparator arms should also be estimated in each period, in addition to the differences in variance and skewness across arms. In addition, we presume that permanent disability is fixed over time and that any variation in it could be represented using additional time-series variation in ${\ell}_{n}^{*}$ and ${t}_{n}^{*}$.

20 This formulation presumes that QoL improvements follow or coincide with life expectancy gains. In cases where QoL improvements precede life expectancy gains, ${p}_{n}$ and ${\Pi }_{n}$ should instead measure pre-treatment life expectancy.
